# Data for Room Fire Model Comparisons

**DOI:** 10.6028/jres.096.022

**Published:** 1991

**Authors:** Richard D. Peacock, Sanford Davis, Vytenis Babrauskas

**Affiliations:** National Institute of Standards and Technology, Gaithersburg, MD 20899

**Keywords:** accuracy assessment, data analysis, experiments, fire models, fire tests, instruments

## Abstract

With the development of models to predict fire growth and spread in buildings, there has been a concomitant evolution in the measurement and analysis of experimental data in real-scale fires. This report presents the types of analyses that can be used to examine large-scale room fire test data to prepare the data for comparison with zone-based fire models. Five sets of experimental data which can be used to test the limits of a typical two-zone fire model are detailed. A standard set of nomenclature describing the geometry of the building and the quantities measured in each experiment is presented. Availability of ancillary data (such as smaller-scale test results) is included. These descriptions, along with the data (available in computer-readable form) should allow comparisons between the experiment and model predictions. The base of experimental data ranges in complexity from one room tests with individual furniture items to a series of tests conducted in a multiple story hotel equipped with a zoned smoke control system.

## 1. Introduction and Background

Analytical models for predicting fire behavior have been evolving within the fire research community for some years. Individuals have tried to describe in mathematical language the various phenomena which have been observed in fire growth and spread. These separate representations often describe only a small part of a fire experience. When combined, they create a complex computer code intended to give an estimate of expected behavior based upon given input parameters. These analytical models have progressed to the point of providing predictions of fire behavior. However, it is important to be able to state with confidence how close are the actual conditions to those predicted by the model.

The Building and Fire Research Laboratory (BFRL) has a program to develop a generic methodology for the evaluation and accuracy assessment of fire models. Our goal is to define a mechanism by which the model predictions can be assessed so that a model user can test the limits of the model predictions. A key aspect of this process is the availability of a sufficient quantity of experimental data with which to compare the performance of any given model. This report presents such a set of experimental data gathered from several sources which can be used to test the limits of a typical two-zone fire model. All of these data are available in computer readable form from the authors. The format of the data has been previously documented [1].

- The remainder of this section provides a *brief* historical perspective of room fire testing leading up to tests specifically designed for comparison with predictive computer models.
- Section 2 describes the process for assessing the accuracy of a predictive computer model. This report details one aspect of this process.
- Sections 3 and 4 present the types of analyses that can be used to examine large-scale room fire test data to prepare the data for comparison with zone-based fire models. Although not every technique was used for all data sets presented in this report, section 3 can be used for guidance in the design of future experiments. In addition, a rough guideline used to judge the quality of the data in each data set is described.
- In sections 5 to 9, five sets of experimental data are detailed. A standard set of nomenclature describing the geometry of the building and the quantities measured in each experiment is presented. Availability of ancillary data (such as smaller-scale test results) is included. These descriptions, along with the data should allow comparisons between the experiment and model predictions.

### 1.1 Early Developments in Room Fire Testing

Before the mid-1970s there was not much need to make experimental studies of the details of room fires. Room fire experiments were typically conducted as an adjunct to studying fire endurance [2,3]. For these experiments, it was necessary to track the average room temperature. This temperature was viewed as the prerequisite for determining the fire exposure of the room structure. Neither the heat release rate nor other aspects of the room fire, such as gas production rates, were of major interest. While as early as 1950, some investigators, conducting full-scale house burns, tried to study the gas production rates to determine how soon untenable environments might exist [4]. There was little incentive to pursue the topic quantitatively. Incentives came with the development of mathematical theories of room fires. Post flashover room fire theories were being developed throughout the 1950s, 1960s, and 1970s. The more detailed understanding necessary for the pre-flashover portion of room fires was becoming achievable by around 1975.

During the 1970s, however, empirical room fire tests were regularly being conducted at many fire research and testing facilities throughout the world. Instrumentation typically included a multiplicity of thermocouples; several probes where gas samples were extracted; smoke meters, typically located at several heights along an open burn room doorway; heat flux meters located in the walls of the burn room; and, possibly, a load platform. The load platform might register the weight of a single burning item, but was of little use when fully-furnished rooms were tested.

Despite the basic role of heat release rate in the room fire, there was no technique available to measure it. Since neither the burning item’s mass loss rate nor the air and gas flows could, in most instances, be determined, the measurements of gas and smoke concentrations at isolated measuring stations were not of much use in tracking species evolution rates.

Even before the era of heat-release-rate focused studies could begin, there were at least three series of notably thorough room fire experiments. Two were conducted at Factory Mutual Research Corporation (FMRC), while a third one was at NBS (former name of NIST). The first series at FMRC [5–7] served as a basis for the Harvard Computer Fire Code. Three replicate full-scale bedroom fire tests, in which the fire grew from a small ignition in the middle of a polyurethane mattress to flashover, were studied in enough detail to define the fire as a series of loosely coupled events. As the components of the fire became better understood, a model of the entire fire growth process as a series of quantitative calculations was developed [8]. To make these tests most useful for a scientific study of fire, several hundred measurements of temperature, radiation level, gas composition, gas velocity, and weight loss were made. The mechanism of fire spread from the initial burning mattress to other room furnishings, estimates of the flow of the gases through room openings, and estimates of the energy balance of the system were all quantified. A second series of tests at FMRC [9] used a simpler test configuration—single slabs of polyurethane foam in the room, instead of fully-furnished bedrooms. A similarly fundamental series of experiments was also conducted at NBS by Quintiere and McCaffrey [10], who examined wood and polyurethane foam cribs burning in well-instrumented rooms. The largest distinction between these tests and earlier test series was the carefully defined purpose to understand the underlying principles of fire growth to be able to predict the progress of a fire in a generic building.

### 1.2 Measurement of Heat Release Rate in Room Fires

**Figure 1:**
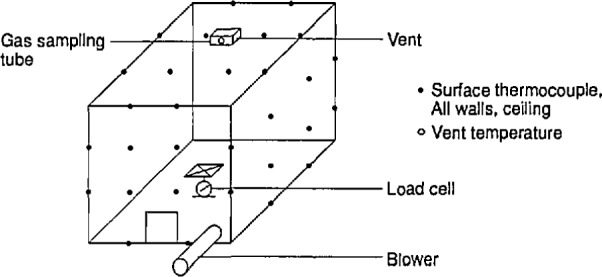
The Monsanto room calorimeter.

The first attempt to develop a technique for measuring rate of heat release in room fires was in 1978, by Fitzgerald, at Monsanto Chemical [11]. He constructed a small room (2.7 m cube) instrumented with a large number of thermocouples, located in the gas space, on the walls, and in the exhaust duct (fig. 1). The room had a forced air supply of 0.19 m^3^/s, from a small 0.15 m square supply duct (later raised to 0.26 m^3^/s [12]), with another duct used to exhaust the combustion products. The room was also equipped with a load cell and a port for extracting gas samples. Fitzgerald realized that a simple measurement of temperatures in the exhaust duct would not be enough to determine the heat release rate. Instead, he developed a purely statistical method—a correlation was sought between contributions from the various temperature measurements to the heat release rate. The stated capacity was 140 kW, which would not now be considered to be full-scale. This system has been sporadically in use at the Southwest Research Institute in San Antonio, Texas. The approach, however, has not been pursued by any other laboratories due to its empirical nature, its limited heat handling capacity, and to concerns about errors due to varying radiative fractions.

**Figure 2:**
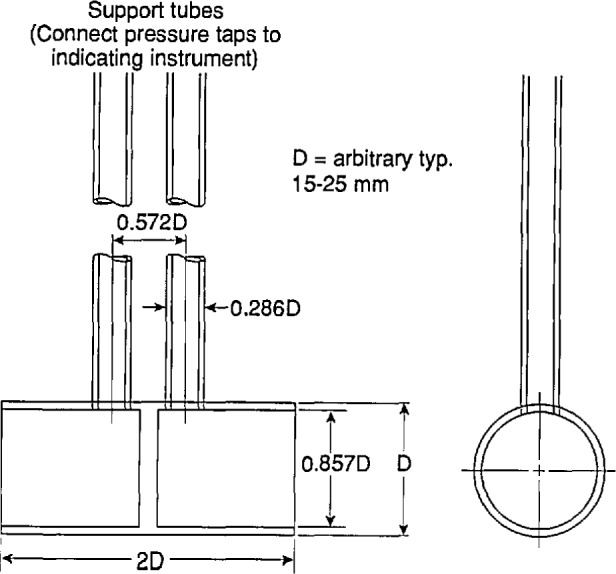
Bi-directional velocity probe.

A sensible-enthalpy calorimeter, such as the Monsanto one, was not judged by the profession to be adequate for the needs. Instead, it was necessary to await the development of two measurement techniques: a robust instrument for measuring the flow rates of air and gas in a soot-laden environment; and a heat release measurement which did not depend on direct measurement of heat flow in inevitably loss-prone systems. The first was developed by Heskestad at FMRC in 1974. Conventional velocity measurement devices are normally precluded from use in fire applications due to several problems. These include clogging of small orifices (an issue with pitot/static probes) and the inability to calibrate properly for high temperature use (hot wire or disc anemometers). The new “bidirectional velocity probe” (fig. 2) solved these problems of measuring air flow rates in rooms, in corridors, and in smoke extraction systems.

By far, the most important development which was needed, however, was the principle of oxygen consumption. As early as 1917, Thornton [14] showed that for many organic fuels, a reasonably constant net amount of heat is released per unit of oxygen consumed for complete combustion. The principles have been covered in detail by Huggett [15] and Parker [16]. The application of this principle to room fires revolutionized the field. Before that, the focus was on point measurements. It is adequate to use measurements of temperatures and other quantities at individual locations in a room as a means of verifying a model if a near-ideal model is already available. Such point measurements, however, were of limited use in developing and extending the models. With the availability of oxygen consumption-based rate of heat release measurements, for the first time quantitative descriptions of fire output could be made.

### 1.3 Standard Room Fire Tests

**Figure 3:**
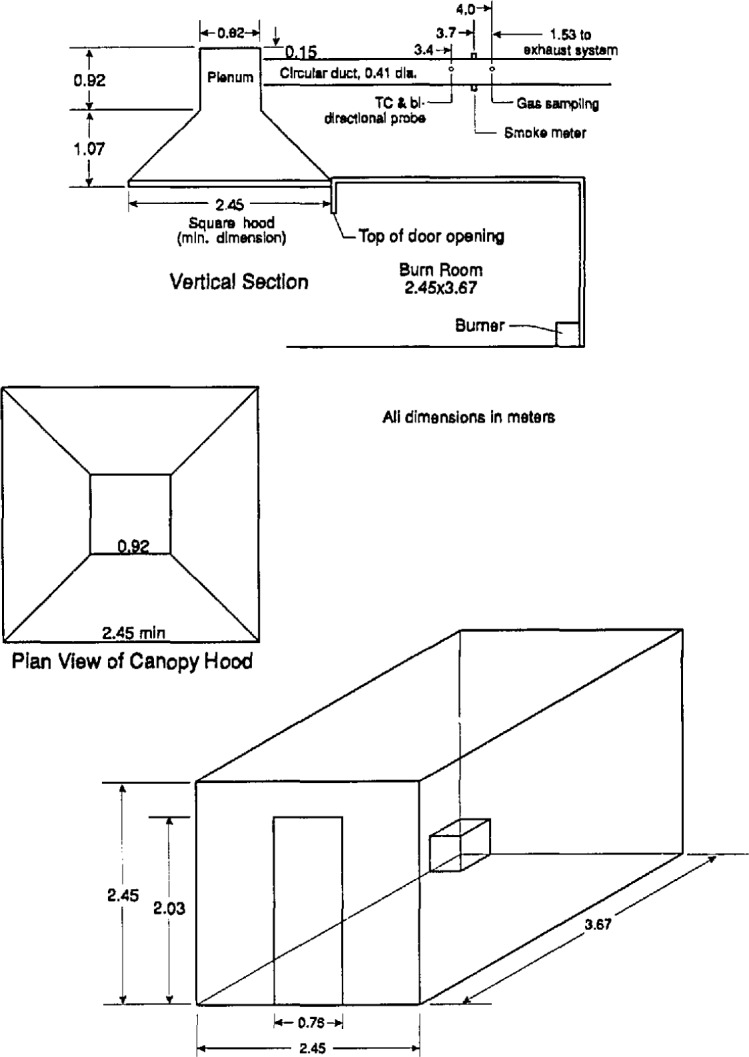
The original (1982) ASTM proposed room fire test.

During the late 1970s and early 1980s several laboratories agreed to develop a standardized method for measuring heat release rates in rooms, based on oxygen consumption. Unlike the Monsanto test, the concern here was in measuring the burning rate of combustible room linings (i.e., wall, ceiling, or floor coverings), and not furniture or other free-standing combustibles. The original development was at the University of California by Fisher and Williamson [17]. Later, extensive development also was done at the laboratories of the Weyerhaeuser Co., and at NBS [18]. The method, in its simplest form, consisted primarily of adding oxygen consumption measurements into the exhaust system attached to a room very similar to that originally used by Castino and coworkers at Underwriters Laboratories [19]. However, they did not measure heat release rates at all. The room was 2.4 × 3.7 × 2.4 m high, with a single doorway opening in one wall, 0.76 × 2.03 m high (fig. 3). The original studies at the University of California led to ASTM issuing in 1977 a Standard Guide for Room Fire Experiments [20]. The Guide did not contain prescriptive details on room size, ignition source, etc., but was simply a guide to good practice in designing room fire tests. ASTM then developed an actual prescriptive test method for room fire tests and published it as a “Proposed method” in 1982 [21]. The 1982 document mandated the above-mentioned room size and also a standard ignition source, which was a gas burner, placed in a rear corner of the room, giving an output of 176 kW. Since the development work at the University of California uncovered problems with a natural convection exhaust system, the actual test specification entailed a requirement to “establish an initial volumetric flow rate of 0.47 m^3^/s through the duct if a forced ventilation system is used, and increase the volume flow rate through the duct to 2.36 m^3^/s when the oxygen content falls below 14 percent.” This specification required a complex exhaust arrangement, and it is not clear that there were many laboratories prepared to meet it. The proposed method was thus withdrawn by ASTM. However, variants of this method continue to be used by several laboratories [22].

**Figure 4:**
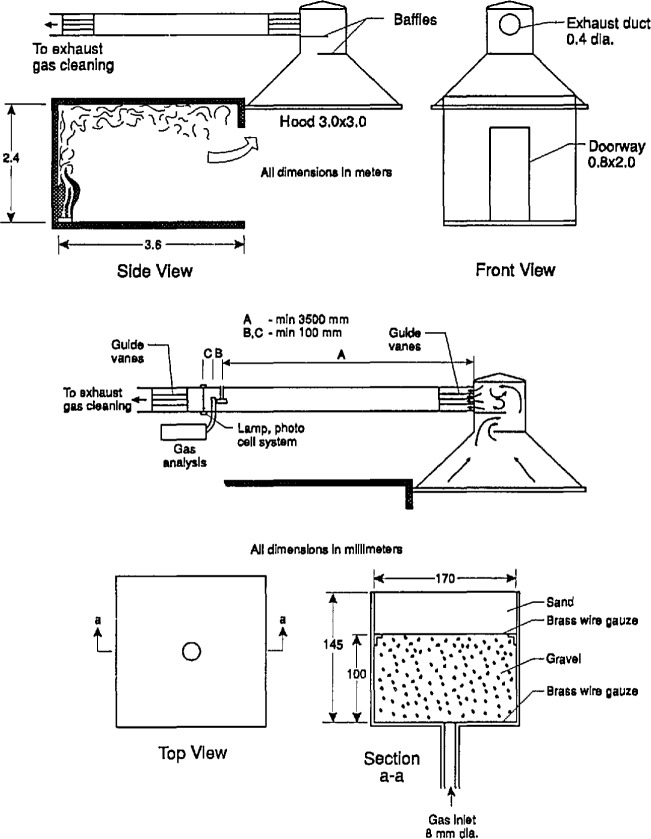
The NORDTEST room fire test.

Following ASTMs disengagement, development of a standard room fire test was accelerated in the Nordic countries, operating under the auspices of the NORDTEST organization. Development was principally pursued in Sweden, at the Statens Provningsanstalt by Sundström [23]. The NORDTEST method [24,25], as eventually published in 1986, uses a room of essentially the ASTM dimensions, 2.4 × 3.6 × 2.4 m high, with an 0.8 × 2.0 m doorway opening (fig. 4). The exhaust system flow capability was raised to 4.0 kg/s, with the capability to go down to 0.5 kg/s to increase the resolution during the early part of the test.

A special concern in the Nordic countries has been the effect of the igniting burner. A parallel project at the Valtion Teknillinen Tutkimuskeskus (VTT) in Espoo, Finland by Ahonen and coworkers [26] developed data on three burner sizes and three burner outputs. The three burners had top surface sizes of 170 × 170 mm, 305 × 305 mm, and 500 × 500 mm. The energy release rates were 40, 160, and 300 kW, respectively. VTTs reported results were on chipboard room linings. They found no significant differences at all among the burner sizes. The burner output did, of course, make a difference; however, the difference between 40 and 160 kW was much larger than between 160 and 300 kW. The VTT conclusion was that either the 160 or the 300 kW level was acceptable. The NORDTEST method itself has taken an ignition source to be at the 100 kW level. If no ignition is achieved in 10 minutes, the heat output is then raised to 300 kW.

ISO (International Organization for Standardization) has adopted the NORDTEST room fire test and is finalizing the standard [27].

### 1.4 Room Fire Tests for Modeling Comparisons

Several systematic test series have been undertaken specifically to provide data for comparison with model predictions. In other cases, tests in which fire properties have been systematically varied (for various reasons) have been modeled using current computer fire simulations. In the first category are the study of Alpert et al. [28] for a single room connected to a short, open corridor, and that of Cooper et al. [29] or Peacock et al. [30] for gas burner fires in a room-corridor-room configuration. The second category is large, but the works of Quintiere and McCaffrey [10], and Hes-kestad and Hill [31] are particularly detailed.

Cooper et al. [29] report on an experimental study of the dynamics of smoke Filling in realistic, full-scale, multi-room fire scenarios. A major goal of the study was to generate an experimental data base for use in the verification of mathematical fire simulation models. The test space involved 2 or 3 rooms, connected by open doorways. During the study, the areas were partitioned to yield four different configurations. One of the rooms was a burn room containing a methane burner which produced either a constant energy release rate of 25, 100, or 225 kW or a time-varying heat release rate which increased linearly with time from zero at ignition to 300 kW in 600 s. An artificial smoke source near the ceiling of the burn room provided a means for visualizing the descent of the hot layer and the dynamics of the smoke filling process in the various spaces. The development of the hot stratified layers in the various spaces was monitored by vertical arrays of thermocouples and photometers. A layer interface was identified and its position as a function of time was determined. An analysis and discussion of the results including layer interface position, temperature, and doorway pressure differentials is presented. These data were later used by Rockett et al. [32,33] for comparison to a modern predictive fire model.

Quintiere and McCaffrey [10] describe a series of experiments designed to provide a measure of the behavior of cellular plastics in burning conditions related to real life. They experimentally determined the effects of fire size, fuel type, and natural ventilation conditions on the resulting room fire variables, such as temperature, radiant heat flux to room surfaces, burning rate, and air flow rate. This was accomplished by burning up to four cribs made of sugar pine or of a rigid polyurethane foam to provide a range of fire sizes intended to simulate fires representative of small furnishings to chairs of moderate size. Although few replicates were included in the test series, fuel type and quantity, and the room door opening width were varied. The data from these experiments were analyzed in terms of quantities averaged over the peak burning period to yield the conditions for flashover in terms of fuel type, fuel amount, and doorway width. The data collected were to serve as a basis for assessing the accuracy of a mathematical model of fire growth from burning cribs.

Heskestad and Hill [31] performed a series of 60 fire tests in a room/corridor configuration to establish accuracy assessment data for theoretical fire models of multi-room fire situations with particular emphasis on health care facilities. With steady state and growing fires from 56 kW to 2 MW, measurements of gas temperatures, ceiling temperatures, smoke optical densities, concentrations of CO, CO_2_, and O_2_, gas velocities, and pressure differentials were made. Various combinations of fire size, door opening size, window opening size, and ventilation were studied. In order to increase the number of combinations, only a few replicates of several of the individual test configurations were performed.

## 2. Assessing the Accuracy of Room Fire Models

In essence, every experiment is an attempt to verify a model. In the simplest case, the model is a hypothesis which is based on some observed phenomenon—or even a single observation — and raises the question “why?” The hypothesis then needs to be tested to determine whether the observation is repeatable and to help define the boundaries of the validity of the hypothesis. In as simple a case as presented here, a yes or no answer may suffice to test the agreement between the model and experiment. For more complex models, the question is not does the model agree with experiment, but rather how close does the model come to the experiment over time. A quantification of the degree of agreement between a model and perhaps many experiments is the subject of the model accuracy assessment process. Quantification is made complicated by the transient nature of fires. Not only must a model be accurate at any point in time, but also have verisimilitude with the rate of change.

### 2.1 Documentation of the Model

For an analytical model designed for predicting fire behavior, the process of accuracy assessment is similar to the single observation case above, but perhaps more extensive because of the complexity of the model. The first step in the process is thorough documentation of the model so other modelers can use it and so its testing can be properly designed. The basic structure of the model, including the limitations, boundary conditions, and fundamental assumptions must be clearly described. Additionally, the functional form of the input parameters must be well-defined to allow any experiments carried out in the accuracy assessment process to be properly simulated (what are the inputs; what are the appropriate units for each). The same applies to the model outputs. In this way, the format of the experimental input and output can be defined to match that of the model.

### 2.2 Sensitivity Analysis

The sensitivity analysis of a model is a quantitative study of how changes in the model parameters affect the results generated by the model. The parameters through which the model is studied consist of those variables which are external to the program, (i.e., input variables), those variables which are internal to the program, (i.e., encoded in the program), and the assumptions, logic, structure, and computational procedures of the model. For this discussion, the model will be considered to be defined by its assumptions, logic, structure, and computational procedures and its sensitivity will be measured in terms of its external and internal variables. The key questions of interest to be investigated by the analyst are: 1) what are the dominant variables? 2) what is the possible range of the result for a given input that may arise from uncertainties within the model? and 3) for a given range of an input variable, what is the expected range for the result?

Sensitivity analysis of a model is not a simple task. Fire models typically have numerous input parameters and generate numerous output responses which extend over the simulation time. So multiple output variables must each be examined over numerous points in time. To examine such a model, many (likely to be more than 100) computer runs of the model must be made and analyzed. Thus, if the model is expensive to run or if time is limited, a full analysis is not feasible and the set of variables selected for study must be reduced. When the set of variables to be investigated must be reduced, a “pre-analysis” for the important variables can be performed or the important variables can be selected by experienced practitioners.

Classical sensitivity analysis examines the partial derivatives of the underlying equations behind a model with respect to its variables in some local region of interest. A complex model may be sensitive to changes in a variable in one region while insensitive in another region. In addition, it is most likely to be unfeasible to determine the intervals for each variable for which a complex model is sensitive. This suggests that stating a single value as a measure of sensitivity is not always sufficient and, consequently, some measure of its variability should be determined to make a global statement of how sensitive a model is to a variable.

Several methods for estimating the sensitivity of a model to its variables are available, each with its advantages and disadvantages. The choice of method is often dependent upon the resources available and the model being analyzed. It is beyond the scope of this paper to go into the details of any of these.

### 2.3 The Experimental Phase

Once an assessment has been made of the relative importance of the model parameters, a selection process is carried out to determine which parameters will be studied in the experimental phase of the accuracy assessment process. Typically, with a fixed budget for model testing, tradeoffs are made in the selection of the number and range of variables to be studied, replication of the experiments, and complexity of the experiments to be performed. Elements of a well-designed experimental program, discussed below, address these trade-offs so the model assessment can be carried out with the available resources.

The number of possible tests, while not being infinite, is large. It is unreasonable to expect all possible tests to be conducted. The need exists to use reason and some form of experimental design strategy to optimize the range of results while minimizing the number of tests. While this is not the forum for a detailed discussion of experimental design, some elaboration is required. Traditionally, a latin-square arrangement or full factorial experimental design is employed to determine the effect of variations in input conditions on output results [35]. This, as expected, results in the number of tests increasing with the number of input variables and variations. However, there exists a reduced factorial experimental plan [36] called fractional replication. The basic concept behind fractional replication is to choose a subgroup of experiments from all possible combinations such that the chosen experiments are representative, amenable to analysis, and provide the maximum amount of information about the model from the number of observations available.

The choice of data to be collected during the experimental phase depends upon the model under evaluation. A description of the input and output data of the model directs the selection of the measurements to be made. The evaluator or test engineer must constrain the range of test conditions to those which apply to the fire model. The test design then includes a varied and representative set of conditions (i.e., enclosure configuration, fuel loading, fuel type, ignition mechanism) from this range.

The evaluator develops the instrumentation design by starting with the model output data and determining suitable algorithms for generating comparable data output from the large-scale tests. This defines the instrumentation requirements, and experience is used to define instrument placement. Unfortunately, any experimental design will include only a fraction of the range of conditions for all the input variables of a complex fire model. The choice of test conditions and instrumentation will, to a large extent, determine the quality and completeness of the accuracy assessment of the chosen model.

### 2.4 Review and Analysis of the Model and Experimental Data

Large-scale tests are performed according to the experimental plan designed by the evaluator. The individual data instrumentation, of which there may be one to two hundred, have to be carefully installed, calibrated, and documented (what they measure and where they are located). Since it is rare to find an individual raw data observation that can be compared to the model output, single data elements are combined to provide derived data which can be compared to the model. Using data collection techniques appropriate to the testing needs, the individual data points are collected and typically processed by computer to provide the desired outputs.

Expected and unexpected uncertainties will define the level of replication necessary for each set of test conditions [37]. There are many sources that can contribute to expected variation in large-scale fire tests, such as variations in the materials or assemblies to be tested, environmental conditions, instruments or apparatus, and calibration techniques used in the measuring process. Because of the nonuniformity of building materials normally encountered and the variability associated with fire exposures and combustion reactions, excellent repeatability is not expected. The development of an experimental plan is, to a large extent, the search for the major factors influencing the outcome of the measurements and the setting of tolerances for their variations [38]. Within the constraints of a fixed budget, replication is usually limited to less than that statistically desired to minimize the unexpected variations. The larger variations that result must be accepted and thus affect the level of confidence in the resulting model accuracy assessment.

As part of the data analysis of the large-scale tests, potential error sources must be quantitatively determined. There are recognized uncertainties in the instrumentation used for each data element as well as random and systematic “noise” in the data acquisition process. The unevenness of burning of a material or the turbulent nature of fluid motion in most fire situations also introduce “noise” into the data analysis process and erratic burning does so among replicate tests. Each step in the data reduction process contributes to the accumulated uncertainties.

Data analysis itself requires the development of a series of algorithms that combine individual data elements to produce the desired output parameter [39]. As can be seen from this short discussion, data analysis of the large-scale tests requires a significant effort before comparisons between the model and the large-scale tests are possible. The size of the data reduction program can be as large and complex as the model being evaluated.

## 3. Analyses Used for the Data

For most large-scale room fire tests, instrumentation is characterized by a multiplicity of thermocouples; several probes where gas samples are extracted; smoke meters, typically located at several heights along doorways or in rooms; heat flux meters located in the walls of the burn room; and, possibly, a load platform. Although certainly useful for evaluation the burning behavior of the specific materials studied, variables representing key physical phenomena are required for comparison with predictive room fire models. Some typical variables of interest from large-scale tests are:
• heat release rate (of fire, through vents, etc.)(W)• interface height(m)• layer temperatures(°C• wall temperatures (inside and out)(°C)• gas concentrations(ppm or %)• species yields(kg/kg)• pressure in room(Pa)• mass flow rate(kg/s)• radiation to the floor(W/m^2^)• mass loss(kg)• mass loss rate(kg/s)• heat of combustion(J/kg)

To obtain these variables, a significant amount of analysis of a large-scale fire test is required. This data analysis requires the development of a series of algorithms that combine individual data elements to produce the desired output parameter. Breese and Peacock [1] have prepared a specially designed computer program for the reduction of full-scale fire test data. In addition to easing the burden of repetitive and similar calculations, the program provides a standard set of algorithms for the analysis of fire test data based upon published research results and a standard form for detailing the calculations to be performed and for examining the results of the calculations. The program combines automated instrument calibrations with more complex, fire-specific calculations such as

- smoke and gas analysis,
- layer temperature and interface position,
- mass loss and flows, and
- rate of heat release.

A description of these algorithms applicable to the analysis of large-scale fire test data is presented below along with an example of each of the algorithms. Although not every one of the techniques was applied to every test (individual measurements available for analysis varied from test to test), many of the techniques were applied to most of the data sets. Details of those applied to an individual data set are available in the sections describing the data sets in sections 5 to 9.

### 3.1 Smoke and Gas Analysis

In the recent past, optical smoke measurements in room fires have been made in several ways:

- vertical or horizontal beams within the room [40],
- vertical or horizontal beams in the doorway [41],
- vertical or horizontal beams in the corridor [42], and
- a diagonal, 45° beam across the doorway plume [43].

The actual measurement is typically made with a collimated light source and directly opposed photometer receiver. This provides a measure of the percentage of the light output by the source that reaches the photometer, and is typically expressed as an extinction coefficient, *k*, as follows:

$$k=\ln\left(\frac{I_{0}}{I}\right).$$ (1)

Bukowski [44] has published a recommended practice for a widely-used design of photometer using an incandescent lamp source. Newer designs [45] are available, however, based on a laser source and are therefore, free of certain measurement errors [46].

Smoke measurements have been reported in a multitude of ways. Many reporting variables suffer from the drawback that the values depend as much on geometric or flow details of the apparatus, as they are on properties of the combustible being burned. Thus, it was important to arrive at a set of variables from which the apparatus influence is removed. There are two such variables. The first is the total smoke *production* for the duration of the test, *P* (m^2^). This variable can be visualized as the area of obscuration that would be caused by the smoke produced in the experiment. The second normalizes the production by the specimen mass loss during burning to form the *yield* of smoke per kg of specimen mass lost (m^2^/kg) [47]. The latter has come to be called the *specific extinction area, σ*_f_. None of the measurement geometries mentioned above, however, are at all useful in characterizing these variables. Such information can be obtained by providing a photometer in the exhaust collection system [48], as, for instance, is done with the ISO/NORDTEST standard (fig. 4). Although such smoke data are sparse, encouraging progress is being made [49].

The specific extinction area is the true measure of the smoke-producing tendency of a material which can be described on a per-mass basis, for instance, wall covering materials. If a fully-furnished room is being tested, or some other configuration is examined where mass loss records are not available, then the smoke production serves to characterize the results.

The total smoke production is computed as

$$P=\int k\dot{V}dt,$$ (2)

where $\dot{V}$ is the *actual* volume flow at the smoke measuring location.

The average specific extinction area is then computed as

$$\sigma_{f}=\frac{P}{m_{0}-m_{\infty }}.$$ (3)

One of primary applications of the yield is in comparing results on the same material conducted in different test apparatus or geometries. Since the effects of specimen size, flow, etc., have been normalized out in this expression, the variable permits actual material properties to be compared.

In some cases, it is also of interest to derive the instantaneous, time-varying expression for *σ*_f_. Its definition is analogous the one given in eq (3).

Gas measurements in the 1970s were typically made by installing probes for CO, CO_2_, etc., analyzers in several places in the room or in the doorway. Data from such measurements had the same limitations as point measurements of temperature: only the behavior at one point was characterized, and no measurement of total fire output was available. Once measurement systems, such as the ISO/NORDTEST room fire test have been adopted which collect all of the combustion products in an exhaust hood, it became a simple manner to instrument that exhaust system for combustion gases.

Old data for gas measurements are typically reported as ppm’s of a particular gas. Similar as to smoke, such measurements depend strongly on the test environment and are not very useful for describing the fuel itself. The appropriate units are very similar to those for smoke. The *production* of a particular gas is simply the total kg of that gas which flowed through the exhaust duct for the duration of the entire test. The *yield* of a particular gas (kg/kg) is the production divided by the total specimen mass lost. As for smoke, there may be scale effects applicable to a particular gas; the yield of a given gas might be expected to be similar for various apparatus and experiments where the specimen was burned under similar combustion conditions [50].

### 3.2 Layer Interface and Temperature

Cooper et al. [29] have presented a method for defining the height of the interface between the relatively hot upper layer and cooler lower layer induced by a fire. Since the calculation depends upon a continuous temperature profile, and a limited number of point-wise measurements are practical, linear interpolation is used to determine temperatures between measured points. The equivalent two zone layer height is the height where the measured air temperature is equal to the temperature *T*_N_ and is determined by comparison of *T*_N_ with the measured temperature profile:

$$T_{N}=C_{N}\left(T_{\max}-T_{b}\right)+T_{b}.$$ (4)

Once the location of the interface has been determined, it is a simple matter to determine an average temperature of the hot and cold layers within the rooms as:

$$\begin{array}{c} T_{u}=\int_{h_{N}}^{h_{t}}\frac{T\left(h\right)}{h_{t}-h_{N}}dh;\\ T_{1}=\int_{h_{b}}^{h_{N}}\frac{T\left(h\right)}{h_{N}-h_{b}}dh. \end{array}$$ (5)

**Figure 5:**
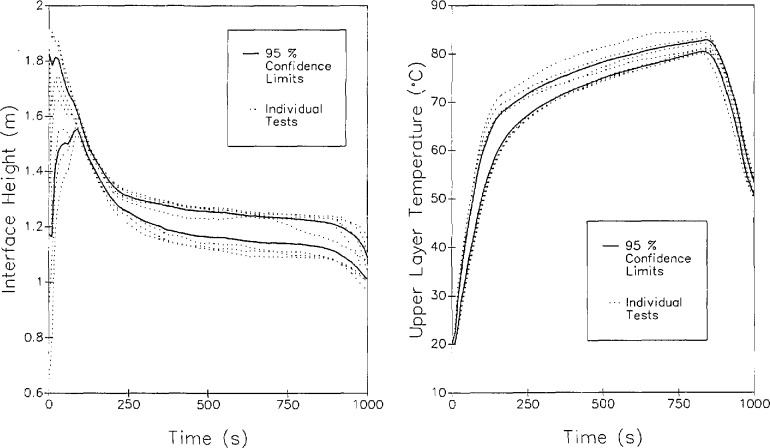
An example of layer interface position and layer temperature calculated from temperature profiles measured during several tests along with estimated repeatability of the measurement [51].

With a discrete vertical profile of temperatures at a given location, the integral can be evaluated numerically. The average layer temperature (either of the lower layer or the upper layer), *T*_avg_, is thus simply an average over the height of the layer from the lower bound, *z*_1_, to the upper bound, *z*_u_, for either the upper or lower layers. Figure 5 shows the results of such a calculation of layer height and layer temperature for a set of eight replicate experiments [51]. Although systematic errors are apparent in the data (two distinct subsets of the data are apparent which may relate to seasonal temperature variations over the testing period) and the limitations inherent in two-zone fire models are equally applicable to these layer height and temperature calculations, the reproducibility of the calculation is good. For a series of large-scale test measurements in a multiple room facility, the uncertainty between 95 percent confidence limits averaged under 16 percent [51].

While the in-room smoke measurement schemes are not useful in quantifying the smoke production or yield, they can be used to deduce the location of the interface in a buoyantly stratified compartment [52]. In this method, if a two zone model is assumed (a smoke-filled upper zone and a clear lower zone), the use of a paired vertical (floor to ceiling) smoke meter and horizontal (near the ceiling) smoke meter can be used to determine the smoke layer thickness. If the smoke layer is homogeneous, *k*_v_/*L*_v_ = *k*_h_/*L*_h_, then the height of the smoke layer *L*_v_ can be given as a simple ratio,

$$L_{v}=h_{t}-\left(\frac{\ln\left(I_{0}/I_{v}\right)}{\ln\left(I_{0}/I_{h}\right)}\right)L_{h}.$$ (6)

where the subscripts v and h refer to the vertical and horizontal measurements.

**Figure 6:**
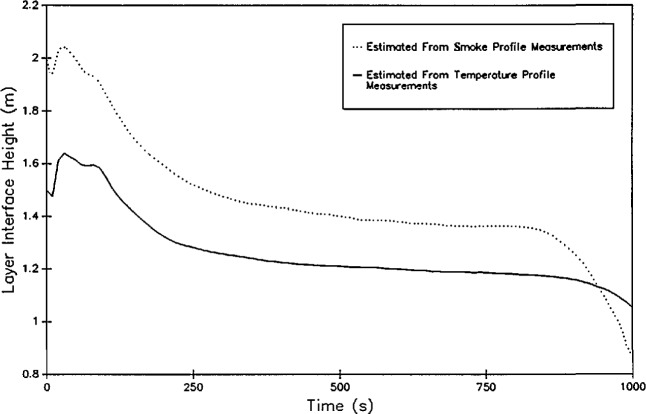
A comparison of hot/cold layer interface position estimated from temperature profiles and from smoke obscuration in one test series (an average of 9 individual tests) [51].

Figure 6 presents a comparison of the smoke layer height calculated from smoke measurements and from temperature measurements for one series of tests [51]. Within experimental uncertainty, the two methods may be equivalent. However, small systematic differences exist. First, the smoke measurement estimates are typically higher than the temperature based calculations. This is consistent with the observations of others, notably Zukoski and Kubota [53], who measured temperature profiles in detail in a scale “room” measuring 0.58 m square with a doorway in one wall measuring 0.43 × 0.18 m. A smoke tracer was used to allow visual observation of the smoke layer thickness along with the temperature profile measurements. They concluded that, since the lower boundary layer is not steady and there are distinct gravity waves along the boundary, the smoke measurements produce a less steep boundary than would be measured from instantaneous profiles at a given instant of time. For tests where the interface height reaches the floor, the temperature based method falters since it is based upon interpolation between adjacent measurement points. Without extensive instrumentation near the floor, a bottom limit at the level of the lowest thermocouple is evident in the temperature-based calculations. However, with the typically higher uncertainty of the smoke-based measurements, the significance of any perceived difference between the two different techniques must be questioned.

### 3.3 Mass Flows

Computation of mass flows through openings can be accomplished through a knowledge of the velocity profile in the opening [54,55]:

$$\begin{array}{c} {\dot{m}}_{u}=C\int_{h_{N}}^{h_{t}}\rho \upsilon W\;dh\;;\\ {\dot{m}}_{1}=C\int_{h_{b}}^{h_{N}}\rho \upsilon W\;dh\;. \end{array}$$ (7)

The velocity profile can be determined in a number of ways. In some experiments, the bi-directional velocity probes described earlier can be used to directly measure velocity in a room doorway. This is usually done by locating 6 to 12 such probes vertically along the centerline of the doorway. Mass flow rates can be computed by eq (7) and can give adequate results for steady-state fires, especially if the opening is much taller than its breadth [56]. Use of such a straightforward technique in non-steady state fires, and especially when the opening is broader than tall, has been shown to give nonsensical results [57]. Lee [56] exploits this method to calculate the mass flow using the pressure drop across the doorway to calculate the velocity. Since the pressure drop across an opening passes through zero as the flow changes direction at the height of the neutral plane, measurement of the pressure profile in a doorway is particularly difficult. Estimation of the pressure in the extreme lower resolution of the instrumentation (as the pressure drop approaches zero) yields an inherently noisy measurement. As such, these measurements are used only as an alternate to the temperature method, to provide an assessment of the consistency of the data collected. As an alternative measurement technique combined with dramatically higher instrumentation costs (several orders of magnitude higher than the temperature measurements), a less detailed profile of measurement points can be used for the pressure profile.

Steckler, Quintiere, and Rinkinen [58] use an integral function of the temperature profile within the opening to calculate the mass flow. Casting their equations in a form that can be used directly to calculate the velocity profile for use in eq (7) yields:

$$\upsilon_{h}=\sqrt{2}g\;T_{d}\int_{h_{N}}^{h}\left(\frac{1}{T_{i}}-\frac{1}{T_{0}}\right)dh.$$ (8)

The temperature profile may also be used with a single pressure measurement to determine the neutral plane height, *h*_N_, required in eq (8). The neutral plane is obtained by solving for *h*_N_ in eq (9) [56]:

$$\begin{array}{l} \Delta p_{b}+\frac{M_{a}p_{a}g}{R}\int_{h_{b}}^{h_{N}}\left(\frac{1}{T_{0}}-\frac{1}{T_{i}}\right)dh=0. \end{array}$$ (9)

**Figure 7:**
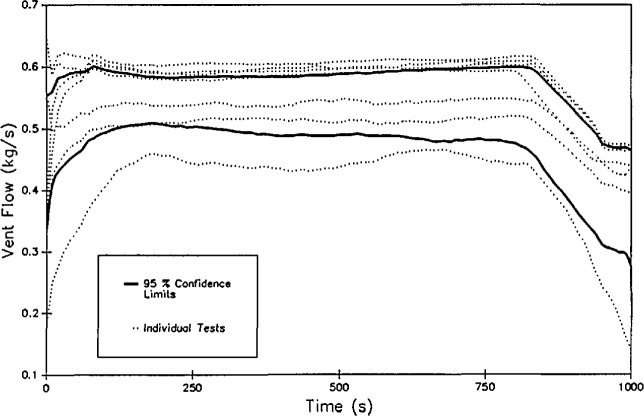
An example of mass flow calculated from temperature profiles measured during several tests along with estimated repeatability of the measurement [51].

Figure 7 shows the results of such a mass flow calculation for a set of eight replicate experiments. For the same set of experiments, the reproducibility of the mass flow calculation is lower than the layer height and temperature calculations, averaging 35 percent [51]. The reasons for this are at least two-fold. The technique used, as described by Steckler et al. [58] was developed for a single room exhausting into an infinite reservoir of ambient air. An extension of the technique for flow between rooms is available [59]. Since the technique depends upon the temperature gradient across the opening as a function of height, the choice of temperature conditions “outside” the opening may be important. Finally, the technique utilizes temperature changes from the neutral plane to the edges of the opening to calculate the flow. Because the smaller temperature change from the neutral plane is in the lower, cooler region, a small variation in temperature should cause more uncertainty in mass flow than in the upper, hotter region where the temperature gradient is larger.

**Figure 8:**
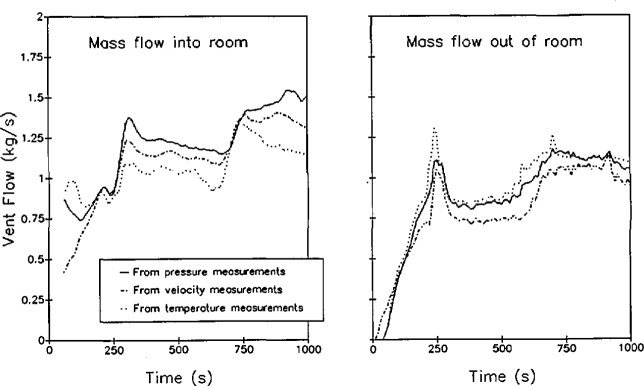
A comparison of calculated mass flow based upon temperature, pressure, and velocity profiles measured during a large-scale fire test [60].

Figure 8 shows a comparison of the mass flow through a typical doorway calculated from pressure measurements, from temperature measurements, and from velocity measurements made in the doorway for a large-scale room fire test [60]. Comparing the mass flow calculations, it is apparent that the temperature based calculations result in a slightly lower calculated mass flow into a room and correspondingly higher mass flow out of a room than for the pressure-based calculations. This is consistent with the difference in calculated neutral plane height for the two methods. As previously discussed, measurement of flows using commercially available pressure transducers is difficult due to the extremely low pressures involved. Compounding the problem for the measurement of the neutral plane height is the desire to know where the flow changes direction. Thus, the most important measurement points are those with the smallest magnitude, just on either side of the neutral plane. Since the neutral plane calculation from pressure measurements searches for the point of zero pressure from the floor up, the calculated point of zero pressure is consistently low.

**Figure 9:**
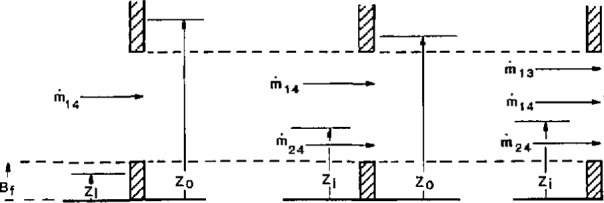
Flow possibilities in a single vent connected to two rooms with different layer boundaries in the rooms [61].

The potential for multiple neutral planes within an opening further complicates the measurement of flow with pressure-based measurements. Jones and Bodart [61] have described an improved fluid transport model with up to three neutral planes within a single opening to incorporate in predictive models (see fig. 9). With potentially different layer boundaries in the two rooms connected to the opening, cross flows are possible between the layers, leading to flow reversals depending upon the relative positions of the two layer boundaries.

Temperature based measurements have far less dependency on the low flow region of the opening, relying on only one pressure measurement near the bottom (or top) of the opening where the pressure gradient is highest. Thus, for the determination of neutral plane height, the temperature based measurement technique seems preferable.

### 3.4 Rate of Heat Release

The large-scale measurement which has benefited the most from the emergence of science in large-scale fire testing is the measurement of the rate of heat released by a fire. With few exceptions [62,63], this is calculated by the use of the oxygen consumption principle. If all the exhaust from a room fire test is collected, measurement of temperature, velocity, and oxygen, carbon dioxide, carbon monoxide, and water vapor concentrations in the exhaust collection hood can be used to estimate the rate of energy production of the fire. With these measurements, the total rate of heat release from the room can be determined from [16]:

$$\begin{array}{l} \dot{q}=\left(E\phi -\left(E_{\mathrm{CO}}-E\right)\frac{1-\phi }{2}\frac{X_{\mathrm{CO}}}{{X_{O}}_{2}}\right)\frac{{M_{O}}_{2}}{M_{a}}{\dot{m}}_{a}\\ \;\left(1-X_{H_{2}O}\right)X_{O2}^{o} \end{array}$$ (10)

where

$$M_{e}=\left(1-X_{H_{2}O}\right)\left(X_{O_{2}}+4X_{{\mathrm{CO}}_{2}}+2.5\right)4+18;$$ (11)

$${\dot{m}}_{e}=C\frac{\sqrt{M_{\text{dry}}\Delta p;}}{M_{e}T_{e}}$$ (12)

$$\frac{{\dot{m}}_{a}}{M_{a}}=\frac{{\dot{m}}_{e}}{M_{e}}\frac{\left(1-X_{H_{2}O}\right)\left(1-X_{O_{2}}-X_{{\mathrm{CO}}_{2}}-X_{\mathrm{CO}}\right)}{\left(1-X_{H_{2}O}^{o}\right)\left(1-X_{O_{2}}^{o}-X_{{\mathrm{CO}}_{2}}^{o}\right)};$$ (13)

$$\phi =\frac{X_{O_{2}}^{o}\left(1-X_{{\mathrm{CO}}_{2}}-X_{\mathrm{CO}}\right)-X_{O_{2}}\left(1-X_{{\mathrm{CO}}_{2}}^{o}\right)}{X_{O_{2}}^{o}\left(1-X_{O_{2}}-X_{{\mathrm{CO}}_{2}}-X_{\mathrm{CO}}\right)}.$$ (14)

Simplifications are available, with some loss of precision, if concentrations of some of the gas species are not measured [64].

**Figure 10:**
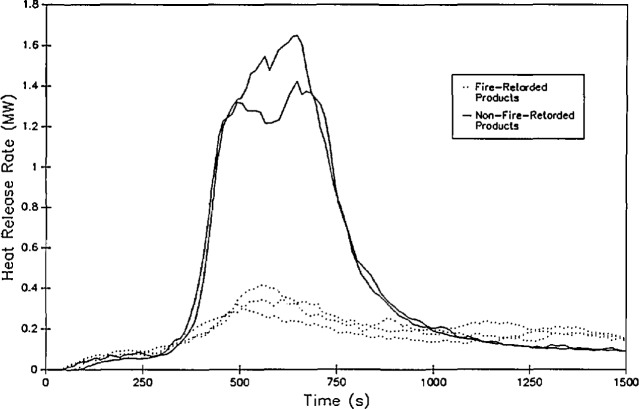
An example of heat release rate calculated from oxygen consumption calorimetry in several large scale fire tests [65].

Figure 10 shows an example of calculated heat release rate from several large scale fire tests [65]. Measurement errors in rate of heat release measurements can be higher than in other measurements, especially for smaller fires. In one study [51], coefficients of variation ranged from 4 to 52 percent. With an oxygen depletion for a 100 kW fire of only 0.26 percent, the calculation of heat release rate suffers the same fate as the calculation of mass flows with pressure probes described above, with much of the uncertainty in the heat release calculations attributable to noise in the underlying measurements.

This technique has been used extensively in both small- and large-scale testing [25,57,66,67]. Babrauskas [57], for instance, has demonstrated the validity of the measurements in a study of upholstered furniture fires. He provides comparisons between replicate tests in the open and enclosed in a room. He notes precision to within 15 percent for fires of 2.5 MW and consistent comparisons of heat release rate expected from mass loss measurements to those measured by oxygen consumption calorimetry.

## 4. Criteria Used to Judge the Quality of the Data

In order to take better advantage of the extensive library of large-scale test data presented in this report, a method of qualifying the data for fast identification was devised. This identification included the type of test that was performed (e.g., furniture calorimeter, multiple room, etc.), the major types of materials tested, the kinds of data available (e.g., gas concentrations, mass flow rates, heat release rate, etc.), and a rating of the quality of the data. This information is presented at the beginning of each section describing the data (secs. 5 to 9).

Since the rating of the data will necessarily be somewhat subjective, a simple type of rating system, one with not-too-fine distinctions, should be employed. The ratings used in this report are the following:

− data not available or not valid or of questionable validity;
±data exist but may not be appropriate for comparison to other tests (check test conditions and quality of data); and
+ data should be appropriate for comparisons.

Availability of small-scale and/or individual burning item data is identified, since these are desirable for development of model input data.

## 5. Single Room with Furniture

This data set describes a series of room fire tests using upholstered furniture items in a room of fixed size but with varying opening sizes and shapes. For the four tests conducted, good agreement was seen in all periods of the room fires, including post-flashover, noting that only fuel-controlled room fires were considered. It was selected for its well characterized and realistic fuel sources in a simple single-room geometry. In addition, the wide variation in opening size should provide challenges for current zone fire models.

### 5.1 Available Data in the Test Series

Following the subjective ratings discussed in section 4, the following set of ratings were apparent from the examination of the test data:
heat release rate (of fire, through vents, etc.)+interface height+layer temperatures+wall temperatures (inside and out)−gas concentrations+species yields±pressure in room−mass flow rate±radiation to the floor+mass loss+mass loss rate+heat of combustion±

In general, the data included in the data set is consistent with the experimental conditions and expected results. Heat release rate, mass loss rate, and species yields are available for all the tests. This should allow straightforward application of most fire models.

### 5.2 General Description of the Test Series

This data set describes a series of room fire tests using upholstered furniture items for comparison with their free burning behavior, previously determined in a furniture calorimeter. Furniture is most often a hazard, not when burned in the open, but rather inside a room [57]. Room fire data lack generality and often cannot be extrapolated to rooms other than the test room; open burning rates have more useful generality. This work was undertaken in a room of fixed size but with varying opening sizes and shapes, in which furniture specimens identical to those previously tested in the furniture calorimeter would be burned. For the four tests conducted, good agreement was seen in all periods of the room fires, including post-flashover, noting that only fuel-controlled room fires were considered.

The conclusions from this study can be summarized as follows:

- The validity of open burning measurements for determining pre-flashover burning rates has been shown for typical upholstered furniture specimens.
- Post-flashover burning of these upholstered items was also seen not to be significantly different from the open-burning rate, for fires which are fuel limited. Fires with ventilation control, by definition, show a lower heat release rate within the room.
- The typical test arrangement of velocity probes spaced along the centerline of the window opening was found to lead to serious errors in computed mass and heat flows. Data taken in the exhaust system collecting the fire products did provide for satisfactory heat release measurements. A method is still lacking which could adequately separate the outside plume combustion heat from that released within the room itself.
- Various relationships for predicting flashover were examined considering the present data. The relationship proposed by Thomas was identified as the most useful, taking into account wall area and properties; however, this relationship may not apply to fires with a very slow build-up rate or for wall materials substantially different from gypsum wallboard.

This program was carried out at the National Institute of Standards and Technology in Gaithersburg, MD in which four experiments were conducted in a single room enclosure; ventilation to the room was provided by window openings of varying sizes. The room was equipped with an instrumented exhaust collection system outside the window opening. The exhaust system could handle fires up to about 7 MW size.

### 5.3 Test Facility

**Figure 11:**
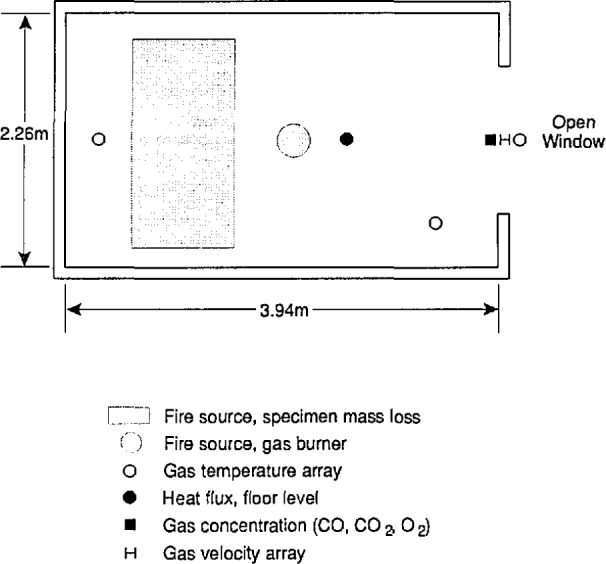
Plan view of experimental room for single room tests with furniture.

**Figure 12:**
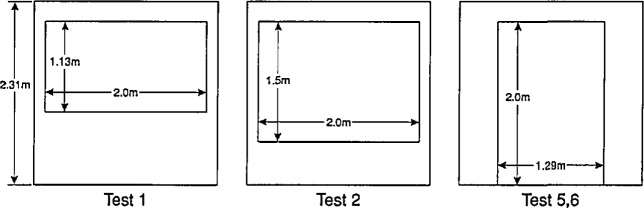
Elevation view of experimental room for single room tests with furniture.

An experimental room with a window opening in one wall was constructed inside the large-scale fire test facility as shown in figure 11. The dimensions of the room and the window openings for the various tests is given in table 1. The soffit depth of the window opening was the same in all cases (fig. 12). For tests 1 and 2, the opening height (and therefore the ventilation parameter $A\sqrt{h}$) only was varied. For test 6, the same $A\sqrt{h}$ was retained but the shape of the opening was changed, compared to test 2. Test 5 resembled test 6, except that the armchair was used. Thus, for specimen type, ventilation factor, and opening aspect ratio, a pair of tests each was provided where these variables were singly varied, the other two being held constant.

The walls and ceiling materials in the room were 16 mm thick Type X gypsum wallboard, furred out on steel studs and joists. Floor construction was normal weight concrete.

The location of the instrumentation used in these experiments is shown in table 2 and figure 11. Two arrays of thermocouples, each consisting of 15 vertically spaced thermocouples, were installed in the room. The top and bottom thermocouples were at the ceiling and on the floor, respectively. In addition, a load cell for mass loss and a Gardon heat flux meter for measuring radiation to the floor were installed on the centerline of the room. Figure 11 also shows the location where a gas burner was used to check the calibration of the exhaust system; the gas burner was removed before testing furniture specimens.

Fifteen closely spaced velocity probes, with companion thermocouples, were located evenly spaced along the vertical centerline to facilitate accurate measurements of mass and heat flow through the opening. Two gas sampling probes were also located along the upper part of the opening center-line.

The exhaust system had an array of velocity probes and thermocouples, together with O_2_, *C*O_2_, and CO measurements to permit heat release to be determined according to the principle of oxygen consumption [13].

### 5.4 Experimental Conditions

Four of the six tests are listed in table 3. The test furniture included a 28.3 kg armchair (F21) and a similar 40.0 kg love seat (F31). Both were of conventional wood frame construction and used polyurethane foam padding, made to minimum California State flammability requirements, and polyolefin fabric. A single piece of test furniture and the igniting wastebasket were the only combustibles in the test room.

The tests in the furniture calorimeter [68,69] made use of a gas burner simulating a wastebasket fire as the ignition source. Because of practical difficulties in installing that burner in the test room, actual wastebasket ignition was used. This involved a small polyethylene wastebasket filled with 12 polyethylene-coated paper milk cartons. Six cartons were placed upright in the wastebasket, while six were torn into six pieces and dropped inside, The total weight of a wastebasket was 285 g, while the 12 cartons together weighed 390 g, for a total weight of 675 g. The gross heat of combustion was measured to be 46.32 kJ/g for the wastebasket and 20.26 kJ/g for the cartons, representing 21.10 MJ in all. Using an estimated correction, this gives a heat content of 19.7 MJ, based on the net heat of combustion. To characterize this ignition source, it seems appropriate to consider a constant mass loss rate $\dot{m}=1.8g/s$ (equivalent to 52.5 kW) for the first 200 s and negligible thereafter.

The test room was conditioned before testing by gas burner fires where the paper facing was burned off the gypsum wallboard and the surface moisture driven off. The room was allowed to cool overnight and between tests.

Initial calibrations with gas burner flows showed adequate agreement, to within 10 to 15 percent, of window inflows and outflows, after an initial transient period of about 30 s. Similarly, during the final, smoldering stages of the furniture fires, a reasonable mass balance was obtained. During peak burning periods in the upholstered furniture tests, such agreement, however, was not obtained.

### 5.5 Examples of Data from the Test Series

**Figure 13:**
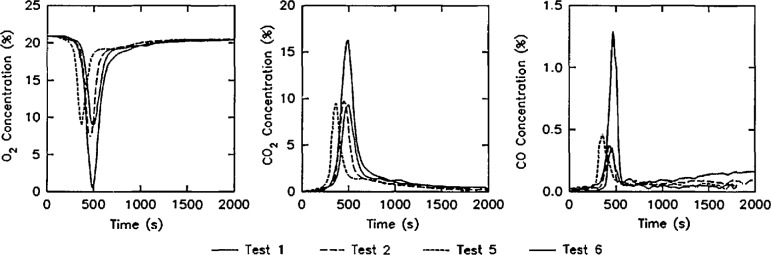
Gas concentrations measured during single room tests with furniture.

**Figure 14:**
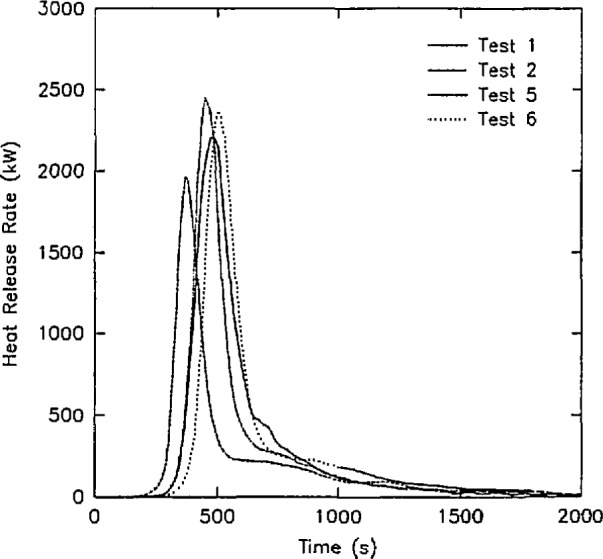
Heat release rate during single room tests with furniture.

**Figure 15:**
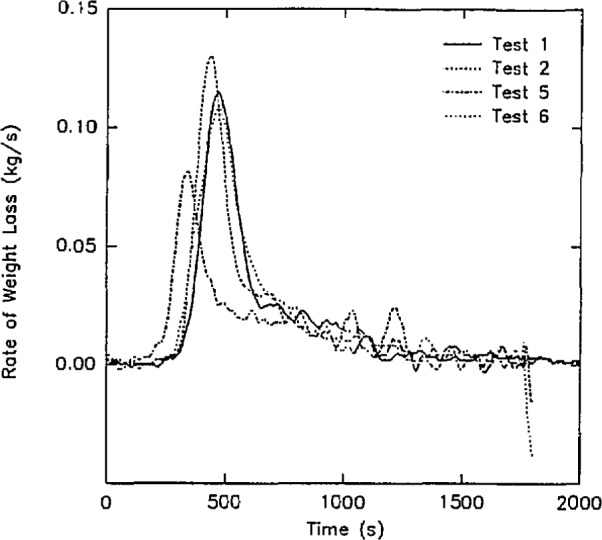
Mass loss rate measured during single room tests with furniture.

Three examples of the data contained in this data set are shown below:

- Concentrations of O_2_, CO_2_, and CO in the upper gas layer in the doorway of the room (fig. 13).
- Rate of heat release from the four room burns in the test series (fig. 14.).
- Rate of mass loss of the burning furniture items for the four room burns in the test series (fig. 15).

In all three of these figures, the consistency of the data set can be seen. In figure 13, the effect of the opening can be seen along with the effect noted above for the heat release rate. For test 1, the O_2_ concentration drops lower (with a concomitant rise in the CO_2_ and CO concentrations) than test 3 or test 6 (the three tests with the same furniture item). However, the three peaks are similar in duration with the fourth peak for test 5 lagging slightly behind. In figure 14, three near replicate curves are seen with a fourth curve of lower peak heat release rate. This is consistent with the two different furniture items burned during the tests. In the original work, Babrauskas [57] suggests an uncertainty of ± 330 kW in these heat release rate measurements. Thus, the three love seat tests (tests 1, 2, and 6) can be considered identical. Not surprisingly, the mass loss rate curves shown in figure 15 shows similar results.

## 6. Single Room with Furniture and Wall Burning

Like the first set, this data set describes a series of single room fire tests using furniture as the fuel source. It expands upon that data set by adding the phenomenon of wall burning in some of the tests. It was chosen for examination because it provides an opportunity 1) to compare burning in the open and in a compartment using the same fuel package, and 2) to compare the effects of non-combustible wall linings versus combustible wall linings in the room [60].

### 6.1 Available Data in the Test Series

Following the subjective ratings discussed in section 4, the following set of ratings were apparent from the examination of the test data:
heat release rate (of fire, through vents, etc.)+interface height+layer temperatures+wall temperatures (inside and out)−gas concentrations+species yields*±*pressure in room+mass flow rate+radiation to the floor*±*mass loss−mass loss rate−heat of combustion−A few notes on the ratings are appropriate. Test 5 in the test series seems to be of questionable quality. It is the only test where the mass flow through the doorway does not exhibit a reasonable mass balance, and although a replicate, radically different than test 2. Thus, its quality must be questioned. Although no mass loss rates were obtained during the tests, the burning materials should allow estimation by the method presented by Babrauskas et. al. [70].

### 6.2 General Description of the Test Series

This data set was chosen for examination because it provides an opportunity 1) to compare burning in the open and in a compartment using the same fuel package, and 2) to compare the effects of non-combustible wall linings versus combustible wall linings in the room [60]. In the former case, the early stages of the fire between the open burns and the room burns are similar; however, it is possible to show how the burning regime changes when influenced by the confines of the room and when the ventilation effects take over. In the latter case, the room wall linings were well-characterized and data are available for estimating heat transfer through the walls. Peak heat release rates as high as 7 MW were measured in these tests.

The relevant conclusions from this study can be summarized as follows:

- Room flashover could occur as early as 233 s with a peak heat release rate of over 2 MW; wood paneling in the room increased the peak heat release to 7 MW.
- The presence or degree of combustibility of a wall behind the bed did not have a significant effect on the free burn rate nor on the smoke and carbon monoxide generation from the furnishing fires. Differences due to the wall were within the experimental scatter found between repeat runs of each test.
- Prior to the ignition of the exposed combustible ceiling surface (paper), the effect of the room on the rate of burning of the furnishings did not appear to be significant. However, subsequent to ceiling surface ignition, noticeable enhancement in the burning rate of furnishings was indicated in all open door room burn tests with one exception.
- Much higher concentrations of carbon monoxide occurred inside the room for a well-ventilated fire than those for a closed room fire. Higher carbon monoxide levels occurred at the 1.5 m height than at the 0.30 m height in the room.
- Mass flow out of the doorway, calculated using three computational techniques, showed good agreement with each other.

### 6.3 Test Facility

**Figure 16:**
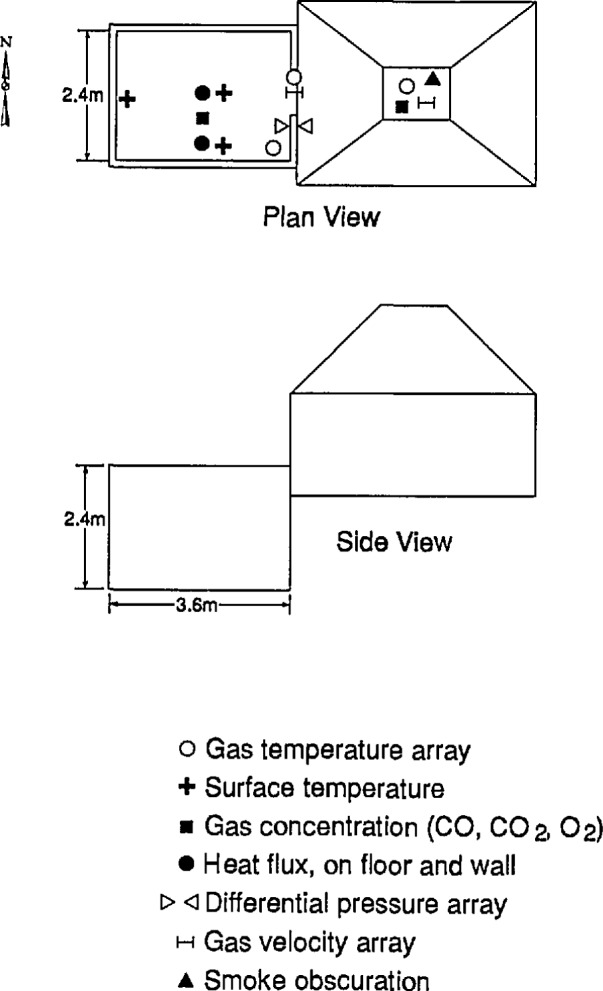
Test room exhaust hood arrangement and instrumentation for single room tests with wall burning.

A furnishing arrangement typical of those in the U.S. Park Service (Dept. of the Interior) lodging facilities was evaluated for its burning characteristics and the times for sprinkler activation. Six open fire tests, i.e., unconfined fires in a large open space, and six room fire tests of one bedroom furnishing arrangement were performed. The test room and exhaust hood arrangement is shown in figure 16. The dimensions of the room and doorway are given in table 4. As can be seen, the 2.44 × 3.66 × 2.44 m high test room was located adjacent to the 3.7 × 4.9 m exhaust collector hood which had an exhaust flow capacity of 3 m^3^/s. In the open burns, the furnishing arrangement was located directly under the hood with the headboard positioned 0.76 m away from the exterior front wall of the room. Two of the open burns had a 2.44 × 2.44 m free standing wall 25.4 mm behind the headboard and in front of the room. This wall was constructed from 12.7 mm gypsum board mounted on 51 × 102 mm steel studs 0.41 m apart. Two other open burns had 6.4 mm plywood lining the same free standing wall. For the room tests, the headboard was located 40 mm away from the back wall. The back and two side walls were 12.7 mm thick gypsum board mounted over 51 × 102 mm steel studs 0.41 m apart. The ceiling was fabricated from 15.9 mm thick fire resistant gypsum board over a sub-layer of 25 mm thick calcium silicate board and was attached to the underside of several steel joists spanning the side walls. The front wall, with a 0.76 × 2.03 m high doorway, was constructed from a single layer of calcium silicate board. Three of the room tests had 6.4 mm plywood over the gypsum board on the two side walls and back wall. In one of the gypsum board lined room tests (test 6), a 0.76 m wide × 2.03 m high and 9.55 mm thick door made from transparent poly(methylmethacrylate) was used for manually closing off the room upon activation of the smoke detector.

Measurements were made in the room and doorway to characterize the fire environment and to allow calculation of the mass flow from the room. These measurements included the air temperature and pressure gradients in the room and air temperature and velocity gradients along the doorway centerline. Total incident heat flux to a horizontal target on the floor was monitored along with the thermal radiance to a vertical surface measured at a height of 0.64 m in the room, next to the left wall, facing the wastebasket. In addition, CO and CO_2_ concentrations were recorded at the 0.30 and 1.5 m heights in the room for test R6. Measurements were also taken in the room to help evaluate sprinkler head and smoke detector responses to the fire environment. Temperatures, velocities, and O_2_ and CO_2_ concentrations in the exhaust gases in the stack were monitored to determine the mass flow through the stack and ${\dot{Q}}_{s},$ the total rate of heat production by the fire.

An average temperature taken across the inlet of the exhaust collection hood was used together with the mass flow in the stack to estimate *h*_s_, the total flux of heat from the fire test room ( ${\dot{Q}}_{s}$minus the heat loss to the room boundaries). The estimated value for the quantity *h*_s_ is actually equal to *h*_s_ minus the heat loss to the surroundings between the room doorway and the inlet to the exhaust collection system. Smoke and CO also were monitored in the stack to help quantify the products of combustion from the room fires.

Location of all instrumentation in the room fires is indicated in table 5 and figure 16. Temperatures in the room and doorway were measured with chromel-alumel thermocouples made with 0.05 mm wire. Because these thermocouples were difficult to prepare and were vulnerable to breakage under normal fire test operations, more robust thermocouples fabricated from 0.51 mm chromel-alumel wires also were employed at these same locations. The larger thermocouples were more susceptible to radiation error and were used primarily as backup measurements. Pressures in the room were measured with probes mounted in one corner of the burn room, flush with the interior surface of the front wall, along the height of the room. Bi-directional velocity probes [71] were employed for measuring the air velocity in the doorway and to note the occurrence of any flow reversal along the doorway. Heat flux was monitored with water-cooled total heat flux meters of the Gardon type. Crumpled newspaper on the floor also was used to indicate if and when the irradiance was sufficient to ignite such light combustible materials in the lower half of the room. Non-dispersive infrared analyzers were used to record the concentrations of CO and CO_2_ in the room and in the stack and oxygen concentration was measured with a paramagnetic type instrument. Stack velocities were measured with pitot-static probes and stack temperatures were monitored with chromel-alumel thermocouples fabricated from 0.51 mm wire. The optical density of the smoke was determined by attenuation of a light beam in the stack. Neutral optical density filters were used to calibrate the light sensor over the range of optical densities from 0.04 to 3.0. At the inlet of the exhaust hood, the average temperature was monitored with a grid of 25 chromel-alumel thermocouples arranged in parallel; each thermocouple was made from 0.51 mm diameter wire.

A sprinkler head with an activation temperature of 71 °C and two different size brass disks, used to simulate faster response sprinkler heads, were used in tests 2 to 6. The smaller disk had a diameter of 9.8 mm, was 0.8 mm thick, and weighed 0.5 g; the larger disk had a diameter of 21.6 mm, was 2.4 mm thick, and weighed 7.3 g. Each disk had a 0.51 mm chromel-alumel thermocouple soldered on its surface. Test R1 did not have a sprinkler or brass disk. The sprinkler in test R3 had a discharge rate of 1.4 L/s (22 gal/min), corresponding to an operating water pressure of 103,400 Pa (15 psi). The other room tests used a dry sprinkler where the pipe was pressured with air to 34,500 Pa (5 psi). In addition, two types of ionization smoke detectors were used in test 6.

### 6.4 Experimental Conditions

**Figure 17:**
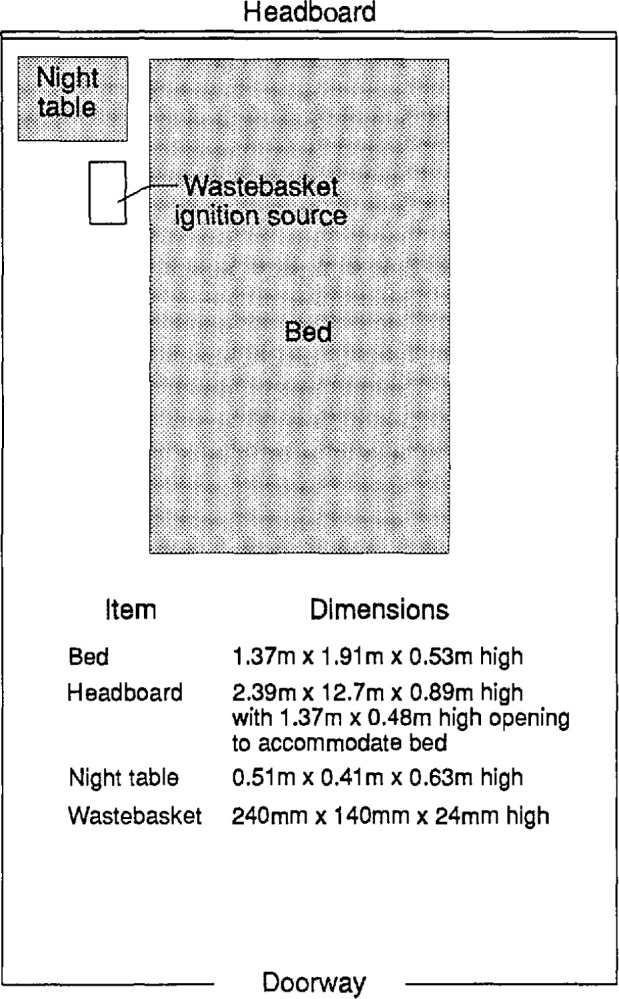
Fire test room arrangement for single room tests with furniture.

These tests are outlined in tables 6 and 7. The standard set of furnishings shown in figure 17 was used for these tests and was based on an inspection of some selected U.S. Park Service lodging facilities at Yosemite National Park in California and at Shenandoah National Park in Virginia. The room furnishings consisted of a 1.37 m wide × 1.91 m long × 0.53 m high double bed, a 2.39 × 0.89 m high headboard, and 0.51 m wide × 0.41 m deep × 0.63 m high night table. Both headboard and night table were fabricated from 12.7 mm thick plywood. The bedding was comprised of two pillows, two pillow cases, two sheets, and one blanket. The pillows had a polypropylene fabric with a polyester filling. The pillow cases and sheets were polyester-cotton. The blanket was acrylic material. The bedding was left in a “slept in” condition which was duplicated to the degree possible in each test. The spring mattress had the same upholstery and padding on the top as on the bottom. The upholstery was a polyester quilted cover. Padding consisted of 6.4 mm polyurethane foam over a fire-retarded cotton felt layer with sub-layers of a cotton felt and a synthetic cellulosic fiber pad. The box spring had a covering of polyester fabric over a layer of cotton felt and a sub-layer of cellulosic fiber pad. Underneath the padding was a wood frame with a steel wire grid on top and a cellulosic cloth cover on the bottom. The combustible weight of each item is given in table 8. The total combustible fire load for this arrangement was 6.0 kg/m^2^ of floor area. With three walls of the room lined with 6.4 mm plywood, the room fire load came to 14.8 kg/m^2^ of floor area.

In all of the tests, the fire was started with match flame ignition of a 0.34 kg (240 × 140 × 240 mm high) wastebasket, filled with 0.41 kg of trash, positioned adjacent to the night table and against the bed. The type and distribution of the contents is shown in table 9.

Duplicate experiments were performed under free-burning conditions (in the open) using three different scenarios: 1) no wall behind the bed, 2) a gypsum wall behind the bed, and 3) a plywood wall behind the bed. In all cases, the same furnishing arrangement and ignition scenario was used.

Three replicate room burns using the same furnishing arrangement and ignition scenario as in the open burns were performed in the room lined with gypsum board and three replicate room burns were carried out in the room lined with plywood. In the latter tests, one fire was extinguished early (167 s) due to sprinkler activation, a second was extinguished after 525 s, and, in the third test, the door to the room was closed after 22 s and reopened after 960 s.

### 6.5 Examples of Data from the Test Series

**Figure 18:**
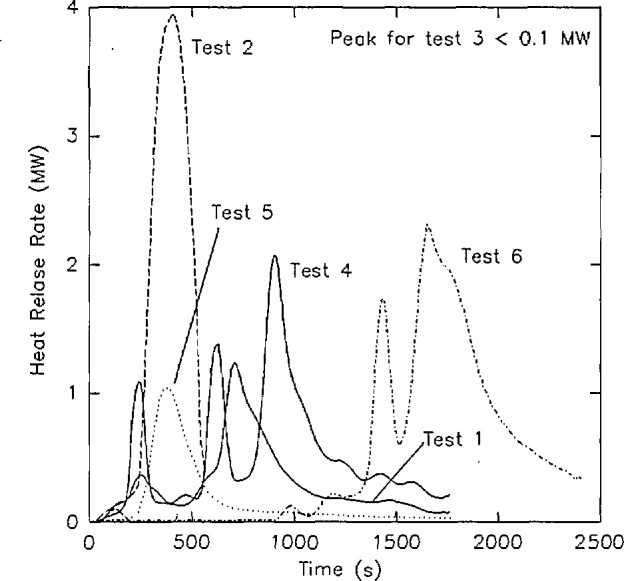
Heat release rate for single room test with furniture and wall burning.

**Figure 19:**
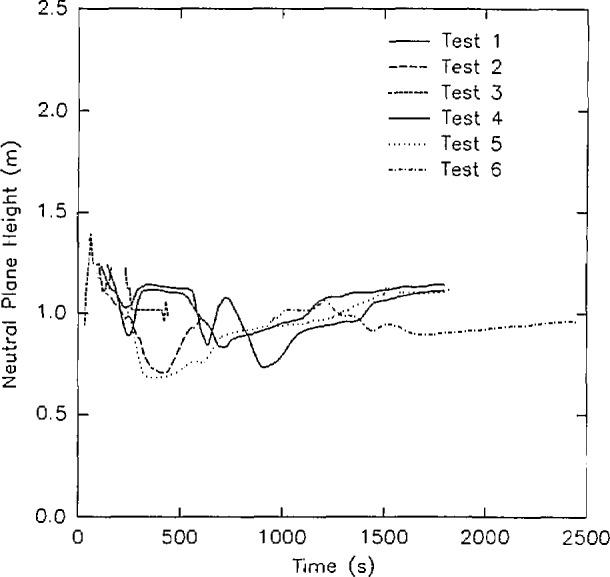
Position of the layer interface for single room tests with furniture and wall burning.

**Figure 20:**
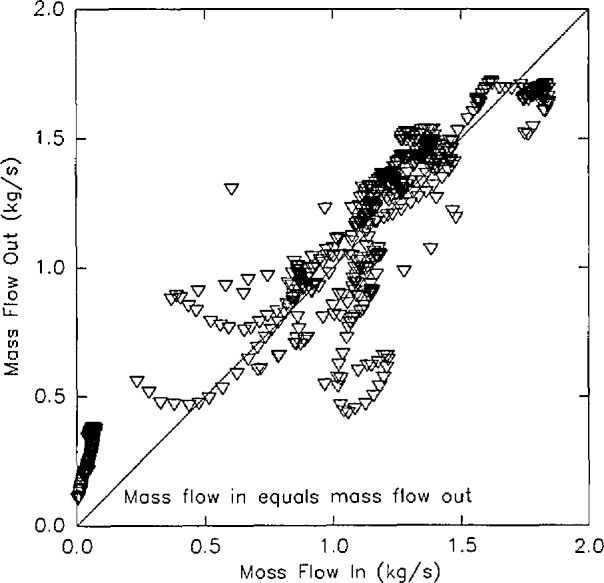
A comparison of mass flow in and mass flow out a doorway during single room tests with furniture and wall burning.

Three examples of the data contained in this data set are shown below:

- Rate of heat release from the six room burns in the test series (fig. 18).
- Position of the interface between the upper and lower layer in the doorway of the room for the six room burns (fig. 19).
- A comparison of mass flow in and out of the doorway for all six tests in the test series (fig. 20).

In figure 18, it is apparent that test 5 is significantly different than test 2, although the two tests are described in the report as replicate tests. In addition, the agreement of the mass flows shown in figure 20 shows an anomaly in the lower left hand corner of the figure also due to test 5. Thus, the quality of that test must be questioned.

The interface position displayed in figure 19 shows two of the three sets of replicates with two pairs of similar curves. Since the doorway was closed during much of test 6, it cannot be considered a replicate of test 4. However, for the two pairs of replicate tests, the agreement is seen as quite good.

Figure 20 shows a comparison of the mass flow in and out of the doorway of the test room. The data for all times and all tests is shown on this figure. Although there is some divergence from the line of perfect agreement, these occur early and late in each test and may be due to the unaccounted for expansion or contraction of the gases within the room. In general, for the hundreds of individual measurements presented, the level of agreement is quite good.

## 7. Three Rooms Including Corridor

This data set describes a series of tests in a multiple room configuration with simple steady-state gas burner fires. It provides a basic set of quantities that are predicted by current fire models for small to medium size fires. Since all fires were gas burner fires, simulation should be straightforward. It is of particular interest since it was undertaken as a part of a program to develop a generic methodology for the evaluation and accuracy assessment of fire models [51].

### 7.1 Available Data in the Test Series

Following the subjective ratings discussed in section 4, the following set of ratings were apparent from the examination of the test data:
heat release rate (of fire, through vents, etc.)+interface height+layer temperatures+wall temperatures (inside and out)*±*gas concentrations*−*species yields*−*pressure in room+mass flow rate+radiation to the floor−mass loss+mass loss rate+heat of combustion+

### 7.2 General Description of the Test Series

This data set is of particular interest because the study was conducted as part of a program to develop a generic methodology for the evaluation and accuracy assessment of fire models. To this end, one specific model (FAST) was chosen because it was well advanced in its development and was fairly well documented by the modeler. A carefully constructed and well-instrumented large-scale fire test facility was developed to provide experimental data for the evaluation of FAST and other models. The parameters studied were selected as the major energy-driven quantities predicted in multi-room fire models. This choice allowed the study to be limited to a manageable set of parameters while providing insight into many of the predictable quantities in room fire models. A three room configuration, with rooms of different sizes, was selected leaving three major variables whose chosen values were combined to define the experiments: fire size, room door opening size, and number of rooms. In total, nine different sets of experiments were conducted, with multiple replicates of each, for a total of 45 tests.

### 7.3 Test Facility

**Figure 21:**
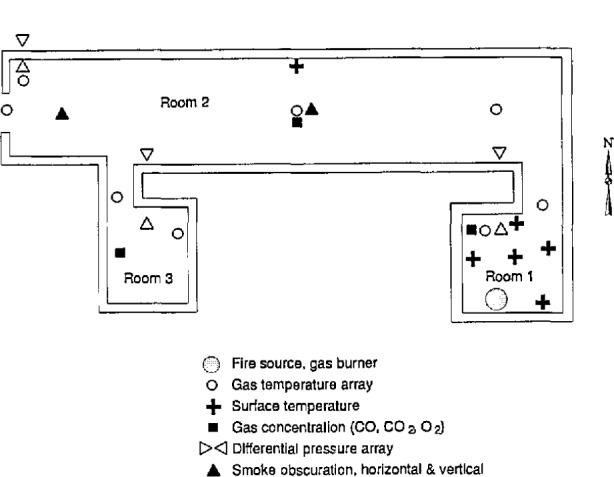
Test room arrangement and instrumentation for three room tests with a corridor.

The experimental arrangement is shown in figure 21. It was a three compartment configuration, with two smaller rooms opening off a corridor 12.4 m long. Table 10 summarizes the dimensions of the three rooms and the connecting vents. The first room, where the fire source was located, had 50 mm thick ceramic fiber insulation under a calcium silicate ceiling and over fire brick walls to reduce thermal losses through these surfaces. The floor of the room was exposed fire brick. The second room ceiling and walls were constructed of steel studding with unfilled stud spaces with gypsum board sheathing and a covering of 13 mm calcium silicate board; this was to assure structural integrity during prolonged exposures to a possible post-flashover fire plume from the door between the first and second rooms. The concrete floor in the second room was covered with 13 mm gypsum board to protect the concrete. The passageway from the second room to the first and third rooms was a small corridor (about 1 m wide ×1 m deep ×2 m high) constructed with the same materials as the second room. Since only warm air circulation was expected in the third room, the walls and ceiling were constructed from 13 mm gypsum board over metal studs, without the calcium silicate covering. The floor was exposed concrete. The construction materials used in this test series, together with their thermophysical properties, are given in table 11. All material properties are literature values and should be considered approximate.

The locations of all instrumentation initially used in the rooms and adjacent exhaust collection hood are summarized in table 12. Some of the instrument locations also are shown in figure 21. Data were recorded with an automatic data logging system at a rate of 24 channels per second.

A 15.9 mm diameter orifice flow meter was used for metering the natural gas flow and a 200 SCFH gas flow meter was used to monitor the acetylene flow.

A 3.7 × 4.9 m hood, having an exhaust flow capacity of about 3 m^3^/s, was located over the doorway from the second room and collected the exhaust from the fire tests. Temperatures, velocities, and O_2_ and CO_2_ concentrations in the exhaust collection hood were monitored with the instrumentation listed in table 12 to determine the rate of energy production of the fire based on oxygen consumption calorimetry.

Part way through the test program, the placement of thermocouples in the first and second rooms were revised to permit greater resolution of the mass flow exhausting from the rooms. At that time, thermocouples were installed also on the unexposed back side of the second room ceiling and north wall to aid in the calculation of the conductive heat losses through these surfaces.

### 7.4 Experimental Conditions

A diffusion flame burner using natural gas, placed snugly against the middle of the back wall of the burn room, served as the fire source. The top of the burner, positioned 0.5 m above the floor, had a 0.34 m square porous ceramic surface with a perimeter of 13 mm wide steel plate. Initially, zinc chloride candles served as the smoke source. Their use was discontinued due to non-uniform time and spatial distributions of the smoke. Later experiments used a mixture of natural gas and acetylene in a heat release ratio of 77 kW of natural gas to 23 kW of acetylene (0.31 g of acetylene per g of natural gas) to achieve a concentration of smoke which provided a visible separation of the hot and cold layers during a test and provided constant smoke production throughout a test.

Gas fires with nominal heat release rates of 100, 300, and 500 kW were conducted under the following configurations:

- second room exit doorway open, third room doorway closed;
- second room exit doorway closed, third room doorway closed;
- second room exit doorway open, third room doorway closed; and
- second room exit doorway closed, third room doorway open.

All the tests are described in tables 13 and 14. Tests 50 K, 100 F, and 100 K had experimental difficulties and were excluded from the tables. For tests 50 D, 300 D, and 300 E, the fuel to the burner was cut off prematurely by the ultraviolet flame sensor.

In the tests with the second room exit doorway closed, a doorway having a realistic 20 mm undercut was used. Unfortunately, measurement or calculation of the flow under the door was difficult. In test 100 O, the undercut in the door was sealed. An opening in the floor, near the door, with an orifice having about the same area as the undercut was used to measure the equivalent flow through the latter.

Tests were initially performed with the data recording system turned on for 300 s before ignition of the gas burner, with the pilot ignited during this 300 s period. Later in the series, a 300 s baseline period, followed by a 300 s pilot flame interval before burner ignition, was also recorded for each test. This allowed an adequate time for the corridor flow behavior associated with the pilot flame to reach steady conditions. The burner was allowed to run for 900 s with data acquisition terminated after recording about 300 s of the cooling period.

To insure a self-consistent definition across the test series and to allow comparison with model predictions beginning at a preset set of conditions, the data from all tests were normalized to a standard definition. Once normalized, the repeatability of a given measurement ranged from excellent to poor. For temperature-based measurements and calculations, the repeatability, as shown by the average standard deviation during steady state burning, was good—typically less than 10 percent of the measured or calculated values. For pressure-based measurements, the repeatability was not nearly as good, at times approaching 70 percent of the values. Much of the disparity between individual tests can be traced to experimental technique, which refined as the testing progressed. The precision of some of the calculations suffers from the propagation of large errors in the individual factors. The rate of heat release or mass flow measurements could be improved by multiple measurements of the same quantity with instruments of different resolution, thus allowing more precise determination of the quantity in the range of interest. For the mass and heat balance calculations, however, such an approach would provide less improvement. Alternate techniques for such determinations should be explored which do not depend as strongly on propagated errors.

### 7.5 Examples of Data from the Test Series

**Figure 22:**
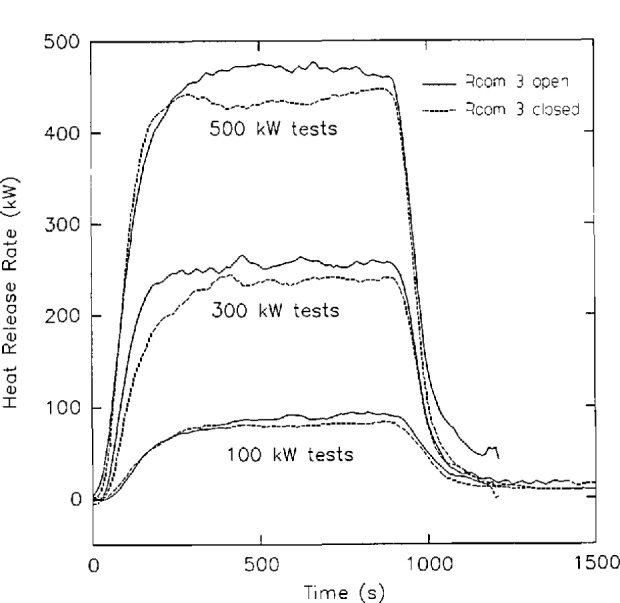
Heat release rate during three room tests with corridor.

**Figure 23:**
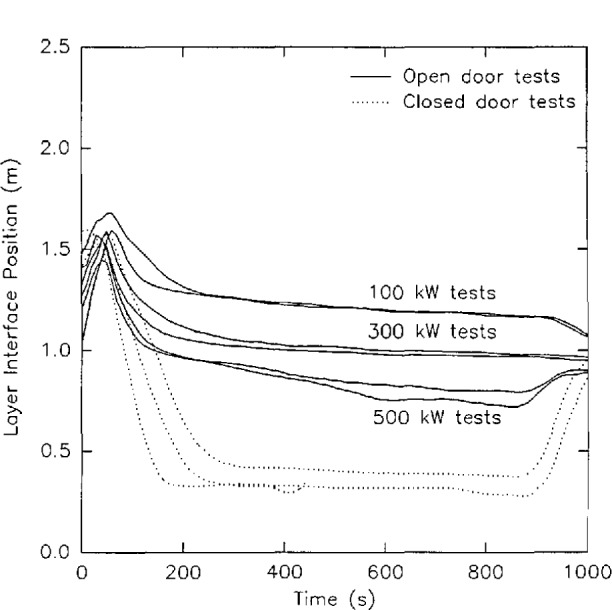
Layer interface heights for tests with three rooms with a corridor.

**Figure 24:**
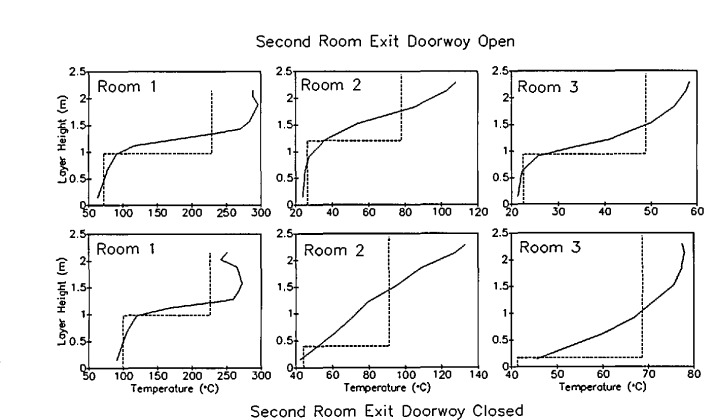
Average temperature profiles for open and closed door tests along with layer temperatures estimated with a two zone assumption.

Three examples of the data contained in this data set are shown below:

- Rate of heat release from the six open door data sets in the test series (fig. 22).
- Interface height for the nine data sets in the test series (fig. 23).
- Vertical profiles of average temperature along with layer temperatures estimated with a two zone assumption (fig. 24).

Figure 22 presents the heat release rate data. These data are only presented for the tests with an open room 2 doorway, since flow through the room 2 doorway to the collection hood was restricted by the closed door, hindering the measurement in the collection hood of the heat release rate of the fire.

Comparing the measured rate of heat release to the heat release calculated from the gas flow rate to the burner (assuming complete combustion of the gas), the measured rate of heat release is consistently low, averaging 19 percent lower than expected. While usually within the experimental uncertainty as exemplified by the average standard deviation for the data sets, the consistently lower readings deserve attention. In the test configuration, flow through the exhaust collection hood is measured minimally downstream from bends in the system. For this reason, accurate measurement of the flow may be suspect.

Figure 23 presents the layer height data. From this figure, the effect of the third room on the layer height in the second room is small, whereas whether the second room exit doorway was open or closed makes a big difference. Comparing similar sets with the door open and closed shows a small time delay in the initial filling of the second room, but with a steady state layer height very similar for the sets with the second room exit doorway in the same position. This result follows logically from the added volume of the third room taking some time to fill, but allowing the second room to fill to the same depth.

Figure 24 shows the average temperature profiles from an open door and a closed door test (both 100 kW tests with room 3 open) overlaid with the upper and lower layer temperature calculated using the two-zone assumption for the test rooms. The solid line represents the average temperature profile; the dotted lines describe the results from the two-zone model. Specifically, the horizontal dotted line shows the height of the layer interface, while the two vertical dotted lines represent the lower and upper layer temperatures and extend through the heights appropriate to these layers. Temperature profiles for room 1, with the burner, are very similar for the open and closed door tests—not surprising, since the door to room 2 is open in both tests. Visually, the two-zone assumption holds better for the open door test than for the closed door test in the cooler rooms 2 and 3. No distinct layering is evident in rooms 2 or 3 in the closed door test. With the closed door, the hot gases come closer to the floor and, along with mixing as the gases reach the end of room 2, lead to a more closely linear temperature profile from the floor to the ceiling. In the test with a closed exit doorway in the second room, mixing occurs at the end of the long corridor in room 2, heating the lower air and cooling the upper air. Even with no distinct break between the layers, interface heights defined using the two-layer assumption show evidence of the mixing with a far more uniform layer thickness in the test with an open doorway in room 2.

## 8. Four Rooms Including Corridor

This data set describes a series of tests conducted in a multiple room configuration with more complex gas burner fires than the previous data set. This study [31] was included because, in many ways, it is similar to the smoke movement study performed at NBS, and permits comparisons between two different laboratories. In addition, it expands upon that data set by providing larger and time-varying gas burner fires in a room-corridor configuration.

### 8.1 Available Data in the Test Series

Following the subjective ratings discussed in section 4, the following set of ratings were apparent from the examination of the test data:
heat release rate (of fire, through vents, etc.)+interface height+layer temperatures+wall temperatures (inside and out)−gas concentrations+species yields+pressure in room+mass flow rate−radiation to the floor−mass loss+mass loss rate+heat of combustion+This data set provides a widely varied set of room configurations and quantities that could be predicted by current fire models for varied fire sizes. Since all fires were gas burner fires, simulation should be straightforward.

### 8.2 General Description of the Test Series

Early enclosure fire tests have been conducted for comparison with fire models involving a single room with natural ventilation through open doors and windows to a large laboratory space. This study was performed to collect data allowing for variations in fire source, ventilation, and geometry, especially for situations with closed doors. This test program was carried out at Factory Mutual Research Corporation (FMRC) in West Glocester, RI, in which 60 fire experiments were conducted in a multiple-room enclosure to furnish validation data for theoretical fire models.

This program was to furnish validation data for theoretical fire models of multiroom fire situations with particular emphasis on health care facilities and no specific conclusions were reached. The data were made available to NBS for further analysis and for making model comparisons.

### 8.3 Test Facility

**Figure 25:**
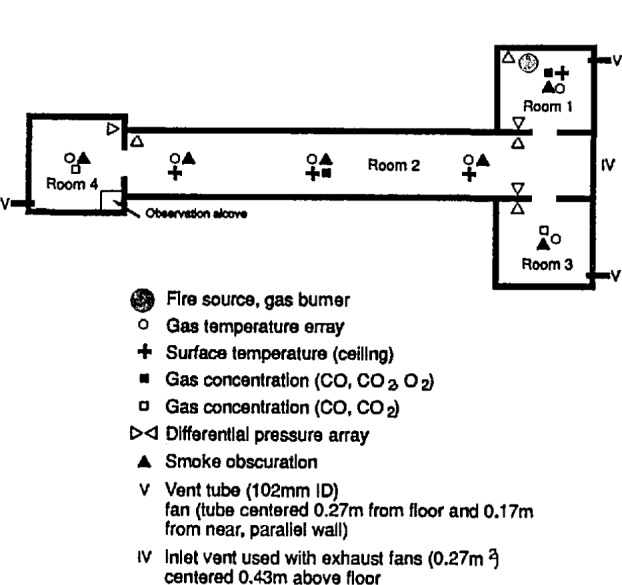
Schematic of facility with instrumentation for four room tests with a corridor.

Figure 25 shows a diagram of the basic facility with indications of instrumentation location. The facility was built on the floor of FMRC’s fire test building, using part of the 67 × 76 m test building where the ceiling height is 18.3 m. The layout in figure 25 shows a burn room and two target rooms connected to a corridor. The corridor was 2.43 m wide × 18.89 m long × 2.43 m high. The burn room measured 3.63 m deep × 3.64 m wide × 2.45 m high; a sealable window opening, measuring 0.85 m square, was centered on the rear wall, 0.34 m down from the top, and a door, measuring 0.92 × 2.05 m high, was centered on the front wall (opening to the corridor). For closed window experiments, the wood-framed Marinite I™ 1 window cover was pressed against a bead of caulking around the steel window frame and held by drop bars positioned into slots on the outside wall.

Room 3, located opposite the burn room, measured 3.65 m deep × 3.64 m wide × 2.45 m high; a door, measuring 0.88 × 2.02 m high, was centered on the front wall (opening to the corridor). Room 4, located at the opposite end of the corridor, measured 3.65 m deep × 3.65 m wide × 2.43 m high and had a 0.88 × 2.02 m high door centered on the front wall (opening to the corridor); an observation alcove, measuring 1.28 × 0.86 × 1.99 m high, was located in the front corner of room 4. Each room was equipped with a 102 mm i.d. vent tube with a 61 mm i.d. orifice meter and thermocouple, with option of exhaust fan (tube centered 0.27 m from the floor and 0.17 m from the closest parallel wall). An inlet vent (0.29 m square) used with exhaust fans was centered 0.43 m above the floor at the end of the corridor between the burn room and room 3. When not in use, the inlet vent was sealed with a gypsum board cover taped in place.

The target room doors were commercial fire doors (wood-faced composite doors with calcium silicate cores, $1\frac{1}{2}$ h rated) mounted on 16 gage steel frames. The burn room door was fabricated from 12.7 mm Marinite I™, mounted in a steel frame lined with Marinite I™. Details of the doors and the spacings (cracks) are given in the original reference [31]. The reader is advised to consult that report if leakage requirements are needed. A summary of the room and door (vent) dimensions is given in table 15.

Gypsum wallboard, 12.7 mm thick, on wood studs was used throughout the experimental facility. In addition, the walls and ceiling of the burn room were overlaid with Marinite I™, also 12.7 mm thick, to harden against repeated fire exposure. The existing concrete floor of the test building was used.

**Figure 26:**
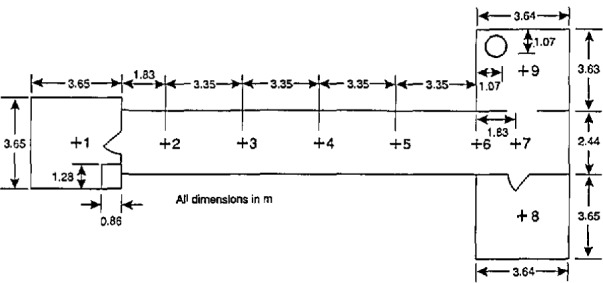
Locations of instrument stations and fire source for four room tests with a corridor.

In figure 26, the instrument clusters, or instrument stations, are numbered 1 through 9. This figure also shows the locations of the instrument stations along with detailed interior dimensions and placement of the fire source. The instrument locations correspond to the vertical thermocouple array of each instrument cluster; locations in the rooms were at the geometric center and, in the corridor, half way between the side walls. These are summarized in table 16.

Gas phase thermocouples were chromel-alumel, 28 gage, insulated with glass braid, except in the burn room where magnesium oxide insulation in 1.6 mm diameter inconel sheathing was used. The wire bundles of the vertical thermocouple arrays entered through the ceiling and were kept taut with steel springs anchored in the concrete floor; the beads extended on horizontal wire a distance of 50 mm to the side of the wire bundles. Chromel-alumel “cement on” thermocouples were used to measure ceiling surface temperatures. All thermocouple wires were connected to 20 gage extension wire immediately outside the enclosure for connections to building signal stations.

The photometers were fabricated according to NBS design [44]. The lamp was operated at an estimated color temperature of 2425 K. The light receiver was a 1P39 phototube with a filter to correct its spectral response approximately to that of the human eye. A 1 m beam length was used and the units in a vertical array were mounted in a rack made from slotted angle framing. The phototube output was transmitted unamplified across a 167 kΩ load resistor to the data acquisition computer. The system response time to sudden blocking of the light beam was about 4 s (to 63 percent attenuation of output). The turbidimeter measured obscuration of light by smoke at three discreet wavelengths, 0.4579 μm (red), 0.6328 μm (blue), and 1.060 μm (infrared). These meters were operated with a beam length of 0.346 m. Response times to sudden obscuration have been estimated at less than 1 ms. The three-wavelength technique can be used to determine smoke particle size and concentration. Responses of photometers and turbidimeters were reduced to smoke extinction coefficient.

The bidirectional flow probe was of standard design [13] with a diameter of 22 mm; it was connected to a Datametrics™ electronic manometer.

Gases were sampled through vertical 12.7 mm o.d. and 9.4 mm i.d. stainless steel tubing, coupled to 12.7 mm o.d. and 9.7 mm i.d. polyethylene tubing above the roof of the enclosure. About 63 m of the polyethylene tubing led from each of the steel sampling tubes to a manifold in the overhead space of the test building, near the gas analyzers, the manifold being exhausted to the atmosphere at a total estimated rate of 4 L/s. Ahead of the manifold, individual sampling lines were joined to the polyethylene sampling tubes for delivering gas samples to respective gas analyzers via glass wool particulate filters, moisture condensers (ice bath), dryers, pumps, and flowmeters. Beckman™ analyzers were employed for oxygen (paramagnetic), carbon monoxide (infrared), and carbon dioxide (infrared). Analyzers were calibrated at the start of each test day. Delay times from the instant of exposure to a constant gas concentration at the open end of a sampling tube to 63 percent of full response of an analyzer were measured at the beginning of the test program and checked at intervals thereafter.

Smoke detectors selected for the program included an ionization type and a photoelectric type. Both types were mounted into separate base units in quick-connect or disconnect fashion. Both the ionization and photoelectric units were designed to alarm when smoke in the detection chamber reached a threshold density. Additionally, the photoelectric unit supposedly had a feature which would lower the threshold for fast rates of rise in smoke density, but this feature may not have been important in this test program.

All pressure differentials from wall pressure taps were recorded on Datametrics™ electronic manometers.

The 102 mm i.d. vent tubes, described above, were 1.63 m long and were provided with an orifice meter and a thermocouple. Pressure differentials across the 61 mm diameter orifice meter, positioned at mid-length and provided with flange connections, were measured with pressure transducers manufactured by Setra Systems™. The thermocouple in each tube was positioned on the tube axis, 0.51 m from the orifice away from the room. Where needed, a Dayton™ fan was coupled to the open end of each vent tube to provide forced exhaust; throttling to desired flow rate was achieved with adhesive tape across the discharge of each fan.

The data were recorded at a rate of one scan per second on the building data acquisition system. The data acquisition computer also controlled the fuel control system where required, i.e., in the automatic growing fire mode. Except thermocouple signals, reduced directly to temperatures by the building data acquisition system, all data reductions were made on computer facilities in FMRC’s Norwood Laboratories from raw data tapes.

### 8.4 Experimental Conditions

Three types of fire sources were used: 1) steady propylene fires at 56 kW on a 0.30 m diameter (sandbox) burner and 522 kW on a 0.91 m diameter burner; 2) propylene fires on the 0.91 m diameter burner programmed under computer control to grow with the square of time, exceeding 1 MW in 1, 2, 4, or 8 min; and 3) a naturally growing fire in a configuration of so-called “Standard Plastic Commodity,” a Factory Mutual test fuel consisting of corrugated boxes with polystyrene tubs in compartments (test 60 only—not discussed in this report).

The 0.91 m diameter, 0.58 m high propylene burner was used for most of the tests. Its design was adopted from D’Souza and McGuire [72] and consisted of a 12 gage steel container with a gas distributor near the bottom, filled with gravel to the 67 percent height, where there was wire mesh screen, and coarse sand to the full height of the burner. The 0.30 m diameter burner was a scaled-down version of similar design. When in use, this burner was placed in a central cavity scooped out of the sand above the screen of the larger burner so its top was level with the top of the larger burner; the sand was back-filled and screeded to present a smooth, unbroken top surface with the burner rims. Ignition was by a propane pilot flame established by a spark at the burner periphery wall before the start of a test; ignition occurred when propylene gas was admitted to the burner. Propylene was selected as a fuel because of its high production of smoke, which made the fire gases easily traceable by the eye and the optical devices.

The 0.91 m diameter propylene burner was calibrated using the FMRC fire products collector located in the fire test building. This device gathers combustion products from a test fire below an inlet cone and then conditions the flow to one of uniform velocity, temperature, and species concentrations. Single-point measurements of temperature and species concentrations, together with the known flow rate, lead to the determinations of heat release, yields of CO and CO_2_, yields of particulates, and flux of optical density.

Total heat release rate $\left({\dot{Q}}_{t}\right)$ was determined from mass flow rate and generation rates of CO_2_ $\left({\dot{m}}_{{\mathrm{CO}}_{2}}\right)$ and CO $\left({\dot{m}}_{\mathrm{CO}}\right)$. Convective heat release rate $\left({\dot{Q}}_{c}\right)$ was determined from mass flow rate and temperature rise. Particulate yield rates $\left({\dot{m}}_{p}\right)$ were established using particle mass concentrations (*C*_p_), together with the mass flow rate. Flux of optical density in the collector was defined as $D_{u}\cdot \dot{\upsilon },$ where *D*_u_ is the optical density (per unit length) and is the volumetric flow rate in the collector duct. Optical density was determined from a photometer of the kind installed in the multi-room enclosure and mounted across the collector duct.

The fuel control for the burners was designed to deliver gas flow to provide energy release rate increments of 32 kW, up to a maximum of 2 MW. Combinations were provided manually with electrical switches, or controlled automatically with the data acquisition computer to generate parabolically growing fires $\left({\dot{Q}}_{t}\propto t^{2}\right)$. The calibrations were performed in the automatic mode. Two different automatic modes were employed, one with a design “growth time” *t*_g_=240 s and one with *t*_g_=480 s. (“Growth time” is the time for a parabolically growing fire to exceed 1 MW.)

**Figure 27:**
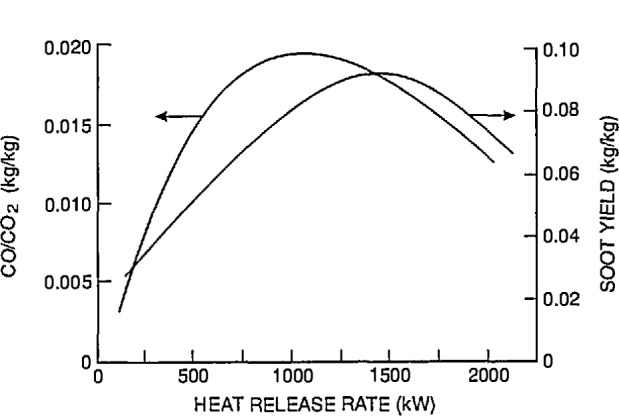
CO, CO_2_, and soot yields from calibration of a 0.91 m gas burner used in four room tests with a corridor.

Calibration results are presented in figure 27. Shown in this figure are the mass generation rate of particulates in ratio to the fuel mass rate and the ratio of generation rates of CO to CO_2_, clearly showing an effect of fire size.

**Figure 28:**
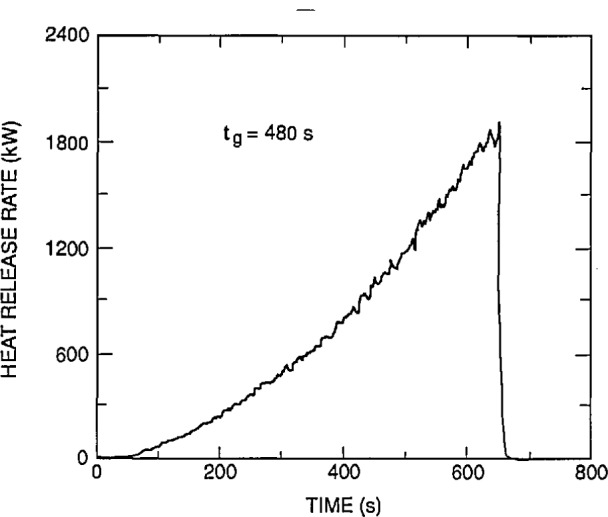
Parabolic fire growth during calibration of 0.91 m diameter propylene burner for four room tests with a corridor.

Figure 28 shows the growth of heat release rate during the calibration run for *t*_g_=480 s. Small drops in gas supply pressure occurred during the run as additional orifices were actuated, resulting in somewhat lower growth rates than would have been the case otherwise. The calibration results discussed in the preceding paragraph have been referenced to a constant gas supply pressure of 274 kPa. During fire tests, deviations of the gas supply pressure from the reference value were reduced by manual adjustments of the pressure regulator for the gas supply.

The 0.31 m diameter propylene burner did not provide enough heat output for accurate measurements in the large fire products collector. Instead, a smaller, but similar, device located at FMRC’s Norwood Laboratories was used. Measurements of yield were made for 1, 2, 3 (1 and 2 flow units in parallel), and 4 nominal flow units. The results are summarized in table 17. It is seen that the total heat release rates per flow unit (FU) average about 27 kW vs 32.6 kW for the larger burner. The convective fraction, ${\dot{Q}}_{c}/{\dot{Q}}_{t},$ averages 0.59, consistent with the result for the larger burner. The mass yield ratios, ${\dot{m}}_{\mathrm{CO}}/{\dot{m}}_{{\mathrm{CO}}_{2}},$ and particulate yields, ${\dot{m}}_{p}/{\dot{m}}_{f},$ may be compared to the results for the larger burner (fig. 27). Only the two-flow unit orifice was used with the smaller burner in the fire tests. An output of 26.9 kW per flow unit is shown in table 17 for this orifice and, hence, the output for the nominal two-flow unit burner will be indicated as 2.10×26.9 = 56 kW.

**Figure 29:**
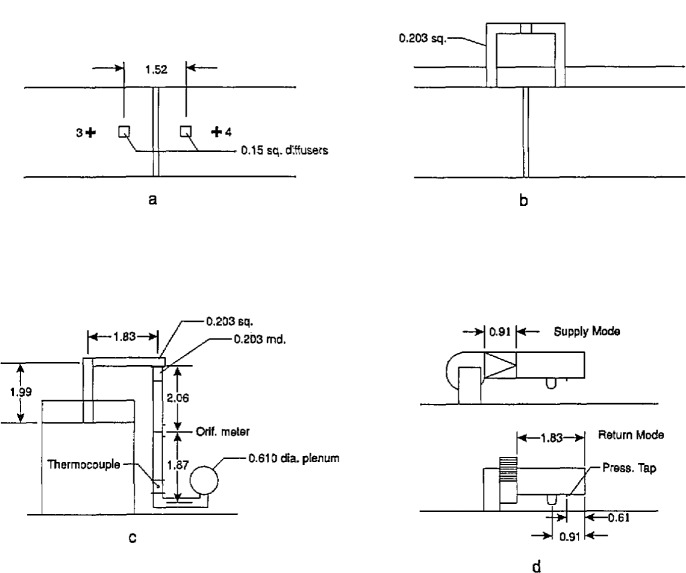
Corridor partition with ventilation ducting for four room tests with corridor.

Starting with test 38, tests were performed to investigate smoke migration in certain ventilation conditions. A sealed partition (fig. 29) was provided between stations 3 and 4 (as identified in fig. 26). Figure 29a is a plan view of the region, showing the instrument stations, the partition (gypsum board on a frame of wood studs with silicone caulking around the perimeter), and the location of ceiling diffusers. As evident in figures 29b and 29c, the diffusers were ducted together into a common horizontal duct, continued as a vertical round tube (aluminum) with an orifice meter (β = 0.6) and thermocouple, followed by horizontal and vertical round ducts (steel) into the wall of a plenum. Figure 29d is a side view of the plenum (steel pipe), showing the entering duct and the inlet of a blower being open to the building space. The blower connection just described (bottom of fig. 29d) was employed when the venting system was in the “return mode,” i.e., with air being drawn into the ceiling diffusers in the corridor. Suction was provided by sub-atmospheric pressures in the plenum developed as a venturi effect by air drawn into the plenum from the building space by the blower; the air flow, suction pressure, and hence the vent flow were controlled with a louvered damper at the exit of the blower. The blower connection shown at the top of figure 29d, with the blower discharge connected via a rectangular-to-round transition to the plenum was used when the venting system was in the “supply mode,” i.e., with air being discharged through the ceiling diffusers into the corridor. The discharge was observed to occur in two oppositely directed ceiling jets, away from the vent, generally aligned with the length of the corridor. At 0.20 m from the vent, the ceiling jets had a maximum depth of about 0.10 m and a width of about 0.4 m. In this case, a positive plenum pressure and the desired vent flow were generated by partially obstructing the open end of the plenum with a plate. Because the blower had to be elevated in this mode, the top section of the vertical round duct in figure 29c had to be replaced with a shorter section.

The blower capacity and plenum were so large that the plenum pressure was practically independent of any flow to and from the corridor ceiling vents. Thus, the plenum may be considered to be a constant pressure plenum in both the return and supply modes, regardless of any fire activity. Total mass venting rates were determined from the pressure differential across the orifice meter together with the flow temperature indicated by the thermocouple. Plenum pressures relative to the atmosphere were determined with a Setra Systems™ transducer connected to the plenum tap indicated in figure 29d.

When the vents were operated in a return mode at approximately 170 g/s total flow with no fire, the plenum pressure was 249 Pa below atmospheric pressure (82 Pa drop in each of the vent ducts before joining and 167 Pa pressure drop in remaining ducting to the plenum, according to experiments). When the vents were operated in a supply mode at approximately 170 g/s total flow with no fire, the plenum pressure was 284 Pa above atmospheric pressure (184 Pa pressure drop from plenum through common ducting and 100 Pa pressure drop in individual ducts to the vents, according to experiments).

A total of 60 tests were conducted over a period of $4\frac{1}{2}$ months. Tables 18, 19, and 20 list the experimental conditions for each test.

The column “HRR” in table 18 indicates the heat release characteristics of the source fires. The entries 56 kW and 522 kW refer to steady total heat release rates at the levels indicated for the 0.30 m diameter and 0.91 m diameter burners, respectively. The entries 60 s, 120 s, 240 s, and 480 s refer to fires growing with the square of time (0.91 m diameter burner), exceeding 1 MW total heat release rate at the indicated growth times.

“Forced vent” indicates whether forced ventilation (exhaust) was provided for the vent tube attached to each of the three rooms. An entry of a number in units of g/s refers to the approximate mass exhaust rate set in each vent tube before each experiment.

The columns “Room 1 door” and “Room 1 window” refer to the dispositions of the door to the burn room and the burn room window, respectively, i.e., open or closed.

Tests 38 to 47 incorporated a partition in the corridor (room 2). The last column in table 20 indicates the mode in which the ceiling vents on either side of the partition were: “None” (ceiling vents blocked); “Natural” (enclosure fires to vent through venting system in response to fire pressures); “170 g/s Ret.” (venting in return mode and set at 170 g/s prior to experiment); and “170 g/s Sup.” (venting in supply mode and set at 170 g/s prior to experiment).

Starting with test 48 (table 19), the corridor partition was removed, the ceiling vents blocked, and the modifications made for accommodating flashover targets in the burn room (not of interest in this report and, consequently, not described).

The smoke detectors were always cleaned with a vacuum cleaner before each test or replaced with a new unit if the preceding test or a pretest with a smoke source (smoldering or flaming paper towel) indicated malfunctions. Starting with the increased heat release rate fires, test 16 onwards, the detectors in the burn room were removed in anticipation of certain destruction in each test.

After test 25, a crack was discovered in the Marinite™ ceiling over the fire source. The affected ceiling area was reinforced with an overlay of 12.7 mm thick Marinite I™, screwed through the existing ceiling into ceiling joists; the overlay measured 1.22 m × 1.65 m.

### 8.5 Examples of Data from the Test Series

**Figure 30:**
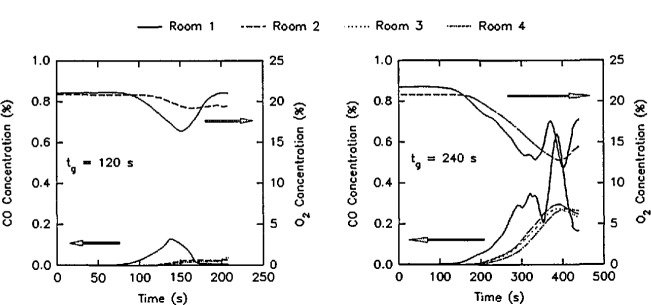
Carbon monoxide and oxygen concentrations in the four rooms during two experiments for four room tests with a corridor.

**Figure 31:**
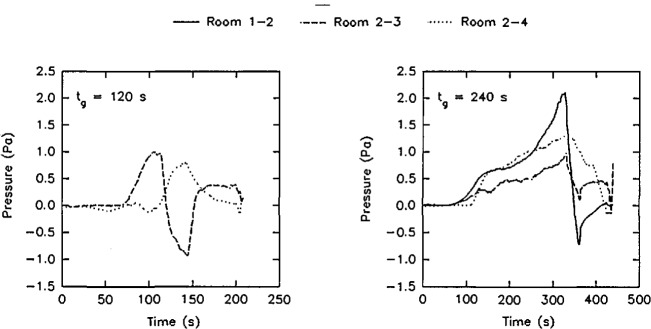
Pressure differences measured in room doorways during two experiments of four room tests with a corridor.

Two examples of data from this test series are shown below:

- CO and O_2_ concentrations in the four rooms of the test structure for two experiments in the test series (fig. 30).
- Pressure differences between rooms of the test structure for two experiments in the test series (fig. 31).

## 9. Multiple-Story Building

By far the most complex set of tests described in this report, this data set describes a series of full-scale experiments conducted to evaluate the current approach to zoned smoke control systems, with and without stairwell pressurization [73]. It was conducted in a seven story hotel with multiple rooms on each floor and a stairwell connecting to all floors. This data set was chosen because it would be considered beyond the scope of most current fire models.

### 9.1 Available Data in the Test Series

Following the subjective ratings discussed in section 4, the following set of ratings were apparent from the examination of the test data:
sheat release rate (of fire, through vents, etc.)−interface height+layer temperatures+wall temperatures (inside and out)−gas concentrations+species yields−pressure in room+mass flow rate−radiation to the floor−mass loss−mass loss rate−heat of combustion−Measured pressure differences between floors of the building and between the stairwell and fire floor of the building are extensive and consistent throughout the test series. For modeling purposes, mass loss rate and heat release rate would have to be estimated. The work of Quintiere and McCaffrey [10] or Babrauskas et. al. [70] could be used to provide such estimates.

### 9.2 General Description of the Test Series

Smoke movement and the performance of smoke control systems were studied in a seven story building with smoke generated from wood fires and from smoke bombs. A total of 12 single experiments were conducted under a variety of conditions: two different fire sizes; sprinklered vs non-sprinklered wood fires; zoned smoke control on or off; stairwell pressurization on or off; with and without ventilation to the outside; and open and closed doors. As expected, in these experiments the zoned smoke control system prevented smoke migration beyond the fire floor.

The relevant conclusions from this study can be summarized as follows:

- For persons trapped above the neutral plane and exposed to smoke from a fire below the neutral plane, the exposure times of concern are of the order of 20 minutes to several hours.
- For fires of this experimental series, the zoned smoke control system effectively maintained positive pressurization around the fire floor.
- The change in mass (d*m/*d*t*) of the gases on the fire floor (or in the smoke zone) can have an adverse effect on smoke control system performance. This effect can be mitigated if the exhaust flow rate is sufficiently large.
- High temperature gases going through a smoke control exhaust fan can result in a significant loss in system pressurization.
- With few exceptions, smoke bombs should not be used for acceptance tests.

### 9.3 Test Facility

**Figure 32:**
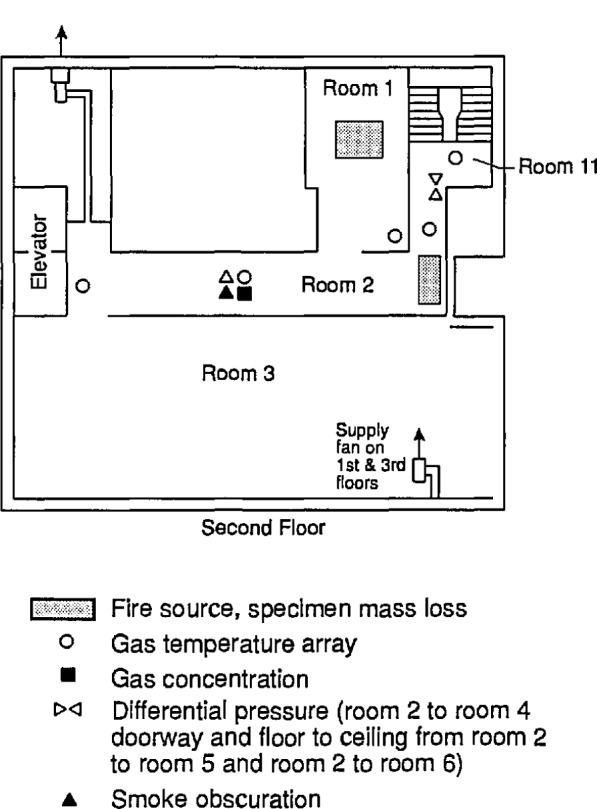
Plan view of one floor for multiple-story building tests (second floor shown).

The Plaza Hotel building was a masonry structure consisting of two wings, one three stories and the other seven stories tall. The two wings were built at different times. The wings were connected to each other at only one location on each floor. The connections between the wings at each floor were sealed off, and the fires were set on the second floor of the seven-story wing (fig. 32), using the shorter wing as an instrumentation area. Areas of the second floor were fire hardened to minimize structural damage to the building. The walls were covered by a 12.7 mm layer of calcium silicate board over a 12.7 mm layer of Type X gypsum board attached to wood furring strips. The ceilings were protected by similar layers of calcium silicate and gypsum board attached to the bottom of the ceiling joists made of commercial steel studs. The floors were protected by calcium silicate board extending about 3 m outward from the fire and by Type X gypsum board for the remainder of the fire hardened areas. The dimensions of the rooms and vents are listed in table 21.

The smoke control systems were designed using the methods presented in the ASHRAE smoke control manual [74], and the design analysis is discussed in detail by Klote [75]. The minimum design pressure difference was 25 Pa (0.10 in H_2_O), meaning that the system should be able to maintain at least this value without a fire. The intent was that the system should function satisfactorily under the most challenging conditions likely to occur during a fire. This level of pressurization is recommended by the National Fire Protection Association [76] for smoke control in unsprinklered buildings. The design pressure difference incorporates the effects of fire in the form of a buoyancy term plus a safety factor, as explained in the Appendix of NFPA 92A. A general discussion of design pressure differences is provided by Klote [77].

In general, the design analysis should be based on likely conditions of open doors and windows; also, the direct effects of the fire must be included in the selection of the minimum design pressure difference. This is the approach evaluated by this project. The design analysis did not include a broken fire room window as one of the likely fire conditions. The importance of this window was not apparent at the start of the project.

In zoned smoke control, the building is divided into a number of zones. These zones may be separate floors, or even a number of floors together. The zone in which the fire occurs is called the smoke zone. For the experiments of this project, each floor of the building was a smoke zone.

Exhausting air from the smoke zone results in air from the outside and from other zones being pulled into the smoke zone. This air flowing into the smoke zone can provide oxygen for the fire. Smoke control systems frequently are designed to exhaust and supply air at six air changes per hour. Most commercial air-conditioning systems are capable of moving about four to six air changes per hour, which probably accounts for the popularity of six air changes in smoke control applications. Current designs are based on the assumption that the adverse effect of supplying oxygen at six air changes per hour is not significant in comparison with the benefit of smoke control. For these reasons, the tests described in this report were conducted at six air changes per hour.

**Figure 33:**
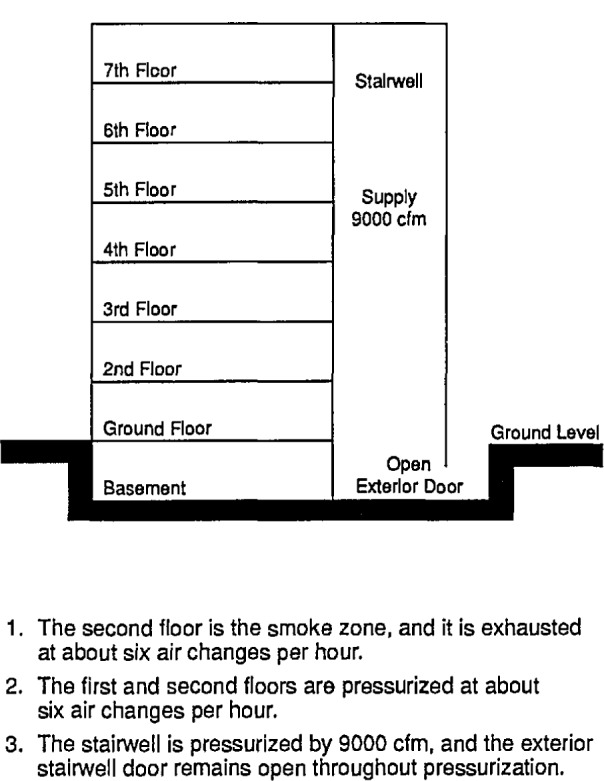
Schematic of the smoke control system for multiple-story building tests.

The Plaza Hotel building had no central forced-air heating, ventilating, and air-conditioning (HVAC) system, so a dedicated system of fans and ducts was installed for zoned smoke control and stairwell pressurization. The smoke control system consisted of the three 0.944 m^3^/s (2000 cfm) centrifugal fans shown in figure 32, plus another centrifugal fan (not shown) located outside and supplying 4.25 m^3^/s (9000 cfm) of pressurization air to the stairwell at the first floor. The smoke control system is illustrated in figure 33. All the test fires were located in the second floor smoke zone. This smoke was exhausted at about six air changes per hour. The first and second floors were pressurized at about six air changes per hour. When the stairwell pressurization system was activated, the exterior stairwell door was open. This approach is intended to minimize fluctuations due to opening and closing doors.

To measure temperatures, pressure differences, gas concentration, smoke obscuration, and wind speed and direction, over 4 km ( $2\frac{1}{2}$ miles) of wire were installed between the instruments and a data acquisition system. The instruments used in this test series are shown in figure 32 and are listed in table 22. All instrumentation channels were recorded at 20 s intervals. More instrumentation was used than was necessary for the evaluation of the effectiveness of a smoke control system with the view that it would be valuable for later computer simulation of the experimental fires.

Temperatures were measured on the fire floor and the other floors at locations shown in figure 32. Additionally, outside air temperature, second floor exhaust fan outlet air temperature, and inlet air temperature of second floor exhaust duct were measured. These temperatures were measured by bare beaded chromel-alumel (type K) thermocouples made from 24 gage (0.51 mm diameter) wire. The wind speed and direction were measured by a propeller-type transducer located 3 m above the roof of the seven story wing.

Smoke meters developed by Bukowski [44] were used to measure light obscuration in the corridors of floors 2, 3, and 7 1.52 m above floor level. This type of meter is an extinction beam consisting of a collimated light source and a detector separated by a path through the smoke. Smoke obscuration is expressed in terms of smoke extinction coefficient.

Carbon monoxide, carbon dioxide, and oxygen were continuously measured at three locations. On floors 2, 3, and 7, gas sampling probes were located at 1.52 m above the floor in the center of the corridor.

Pressure differences were measured by variable reluctance differential pressure transducers.

### 9.4 Experimental Conditions

Smoke movement and the performance of smoke control systems were studied with smoke generated from unsprinklered wood fires, sprinklered wood fires, and smoke bombs. All the windows were closed except for the window of the fire-hardened room during test 12, which was left open to simulate the effect of a broken window. For many of the tests, the second floor stairwell door was cracked open 13 mm, simulating the gap of a door warped due to high differential temperatures. The specific doors open, and other test conditions, are listed in table 23.

**Figure 34:**
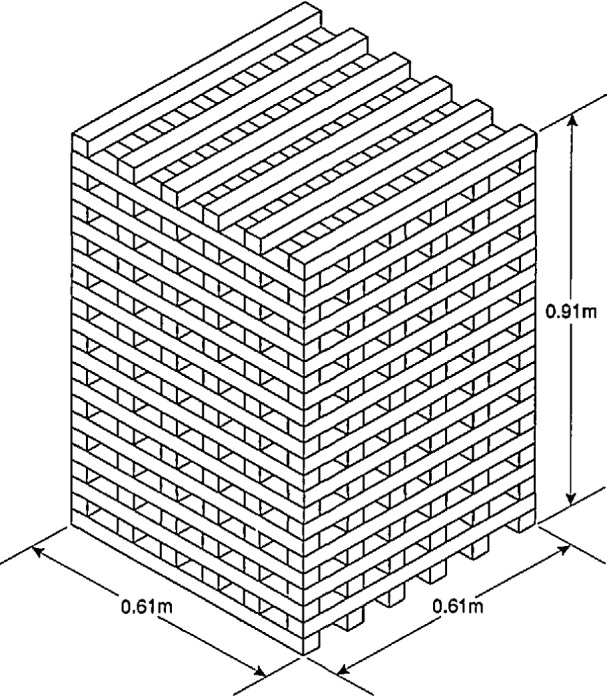
Configuration of nominal 68 kg wood crib used as a fire source for multiple-story building tests.

For the unsprinklered fires, wood sticks were arranged in geometric piles called cribs. The cribs were constructed of fir sticks 38 × 38 mm × 0.61 m long. The sticks were fastened together with 8d common nails. The crib illustrated in figure 34 was 24 layers high and weighed about 68 kg the 24-layer crib was used for most of the tests. The exception was for test 3 in which smaller cribs of 18 layers were used because of concern about possible damage to the building’s structural system.

All of the fires used two cribs located in the second floor corridor (fig. 32), except for test 12 in which four 24-layer cribs were located in the fire-hardened room on the second floor. The cribs were stored in a room in the Plaza Hotel without humidity control. However, the moisture content was measured at less than 6 percent for all the cribs. By extrapolation of data for similar cribs burning in free air [78], it was estimated that two 24-layer cribs would have a peak energy release rate of 1.5 MW, and two 18-layer cribs would have a peak energy release rate of 1.0 MW. Four 24-layer cribs would have an energy release rate of 3.0 MW.

A 0.13 m diameter metal pan with 1 L of heptane was centered under each crib as an ignition source; the heptane was ignited with a propane torch.

The sprinklered fires were set in the corridor, as illustrated in figure 32, and two 24-layer cribs as described above were used. Test 10 was with a listed quick-response pendant sprinkler with a 71 °C operating temperature. Test 11 was with a pendant sprinkler with a fusible element operating at 63 °C and a bimetallic disk for on-off operation opening at 74 °C and closing at 35 °C. The sprinklers were located above the cribs about 0.64 m from the center of the two cribs. The deflector of the quick-response sprinkler was 0.10 m below the ceiling, and the deflector of the on-off sprinkler was 0.15 m below the ceiling. The density of spray was measured by collecting water from the sprinklers in pans located so that the pan tops were at the elevation of the top of the cribs. The quick-response sprinkler produced an average density of 0.21 L/s m^2^ and the on-off head produced an average density of 0.28 L/s m^2^.

The smoke bombs were ignited at the same corridor location as most of the other tests. Three smoke bombs, rated by the manufacturer for a three minute duration, were wired together, placed in a metal container, and ignited.

Because so many complicated detection and activation schemes are in common use, simulation of one particular activation approach would have been of limited value. Thus, it was decided to simulate the extreme conditions of very fast activation and delayed activation. For very fast activation, the smoke control system was activated before ignition for tests 2, 3, 6, 7, and 12. This is considered to be similar to what would happen if the smoke control system were activated rapidly enough so that very little smoke would reach the horizontal barriers of the smoke control system before ignition. A four minute time was arbitrarily selected for the delayed activation for tests 8 and 9.

### 9.5 Examples of Data from the Test Series

**Figure 35:**
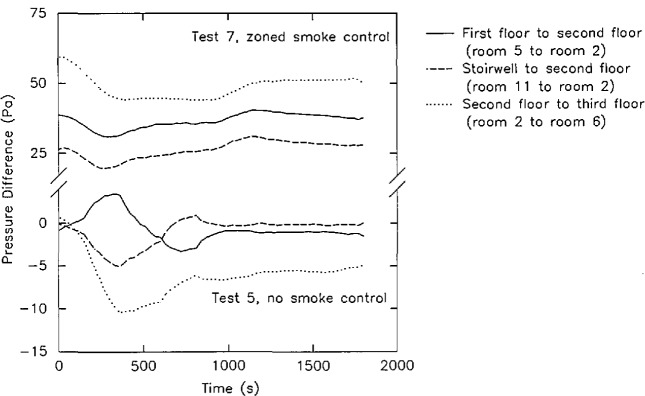
Pressure difference between floors in two multiple-story building tests.

**Figure 36:**
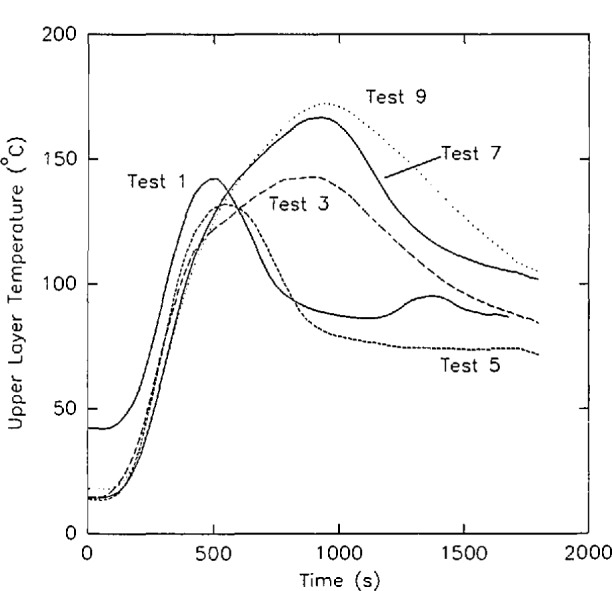
Upper layer temperature during tests in a multiple-story building.

Two examples of data from this test series are shown below:

- Pressure differences between the fire floor and the floors above and below the fire floor for a test with and a test without smoke control (fig. 35).
- Upper layer temperatures for all the experiments in the test series (fig. 36).

Included in the full report of this test series [73] is an analysis of the pressure differences for the tests shown in figure 35. With a simple predictive model developed in the report, calculated pressure differences between the floors agreed quite well with measured values.

## 10. Summary and Conclusions

BFRL has been working to develop a generic methodology for fire model evaluation. This report has presented documentation of more than 125 individual room fire tests that can be used for comparison with zone-based predictive models. Five different test series were included in the discussion:

- A single room test series with furniture and varied opening sizes,
- A single room test series with furniture and wall burning,
- A three room test series including a corridor with multiple replicates of several different experimental conditions,
- A four room test series including a corridor with large growing fires, and
- A multiple-story building test series with a zoned smoke control system.

Derived outputs from individual raw data elements were developed for all the tests in a single, consistent format together with the mathematical treatment used to make the calculations. Geometry of the room(s) and the measurements taken for all the tests were reviewed and presented using the same nomenclature for all tests, simplifying comparison of data from different tests (from different laboratories).

Since the tests to be included in this database were chosen to present a broad range of challenges for the current generation of fire models, the comparisons with current fire models may not always be favorable. In some cases, the tests include physical phenomena not included in some models (such as forced ventilation, flow in long corridors, or multiple stories in a building). Thus this base of data can also be viewed as providing input for model developers to extend the capabilities of the models.

## Figures and Tables

**Table 1:** Room and vent sizes for one room tests with furniture

Location^a^	Room	Vent	Dimensions (m)^b^
Room 1 (burn room)	✓		2.26 × 3.94 × 2.31
Doorway or window, room 1 to ambient		✓	2.0 × 1.13 × 0.31 (test 1)
			2.0 × 1.50 × 0.31 (test 2)
			1.29 × 2.00 × 0.31 (test 5)
			1.29 × 2.00 × 0.31 (test 6)

a Notation used for rooms and vents were changed from the original report to be consistent throughout this report.

b For rooms, dimensions are width × depth × height. For vents, dimensions are width × height × soffit depth.

**Table 2:** Location of instrumentation for one room tests with furniture

Room location^a^	Measurement type^b^	Position^c^
Room 1 (burn room)	Gas temperature arrays in two positions	0.17, 0.33, 0.50, 0.66, 0.83, 0.99, 1.16, 1.32,1,49, 1.65, 1.82, 1.98, and 2.15 m
	Surface temperature in two positions	On floor and ceiling
	Heat flux	On floor
	Specimen mass loss	
Doorway, room 1 to ambient (burn room doorway)	Gas temperature array	See footnote d
	Gas concentration, CO, CO_2_, and O_2_	1.66 and 1.72 m
	Gas velocity array (bi-directional velocity probes)	See footnote d
Exhaust hood	Gas temperature array	Nine positions evenly spaeed
	Gas velocity array	Nine position evenly spaced
	Gas concentration, CO, CO_2_, and O_2_	At centerline of hood
	Smoke obscuration	At eenterline of hood

a Notation used for rooms and vents were changed from the original report to be consistent throughout this report. For reference, names used in the original report are shown in parentheses.

b Notation used for instrumentation was changed from the original report to be consistent throughout this report. For reference, names used in the original report are shown in parentheses.

c Distances are measured from floor.

d 15 locations spaeed evenly from bottom to top of vent^c^.

For test 1: 0.93, 1.00, 1.08, 1.15, 1.22, 1.29, 1.36, 1.43, 1.50, 1.57, 1.64, 1.72, 1.79, 1.86, and 1.93 m.

For test 2: 0.58, 0.67, 0.77, 0.86, 0.96, 1.05, 1.14, 1.24, 1.33, 1.43, 1.52, 1.61, 1.71, 1.80, and 1.90 m.

For tests 5 and 6: 0.13, 0.25, 0.38, 0.50, 0.63, 0.75, 0.88,1.00, 1.13, 1.25, 1.38, 1.50, 1.63, 1.75, and 1.88 m.

**Table 3:** Tests conducted for one room test with furniture

Test	Chair	Soffit depth (m)	Opening width (m)	Opening height (m)	$\begin{array}{c} A\sqrt{h}\\ \left(m^{5/2}\right) \end{array}$
1	love seat	0.31	2.0	1.13	2.43
2	love seat	0.31	2.0	1.50	3.65
5	armchair	0.31	1.29	2.00	3.65
6	love seat	0.31	1.29	2.00	3.65

**Table 4:** Room and vent sizes for one room tests with furniture and wall burning

Location^a^	Room	Vent	Dimensions (m)^b^
Room 1 (burn room)	✓		2.44 × 3.66 × 2.44
Doorway, room 1 to ambient		✓	0.76 × 2.03 × 0.31

a Notation used for rooms and vents were changed from the original report to be consistent throughout this report. For reference, names used in the original report are shown in parentheses.

b For rooms, dimensions are width × depth × height. For vents, dimensions are width × height × soffit depth.

**Table 5:** Location of instrumentation for one room tests with furniture and wall burning

Room location^a^	Measurement type^b^	Position^c^
Room 1 (burn room)	Gas temperature arrays (0.51 mm and 0.05 mm thermocouple trees)	Two sets of each 0.20, 0.41, 0.61, 0.81, 1.02, 1.22, 1.42, 1.63, 1.83, 2.03, and 2.24 m
	Gas temperature	Center of room, 2.34 m
	Surface temperature (thermocouple near brass disks)	On three walls, 2.31 m
	Gas concentration, CO and CO_2_	0.30 and 1.52 m
	Heat flux	On floor and 0.64 m
Doorway, room 1 to ambient (burn room doorway)	Gas temperature arrays (0.51 mm and 0.05 mm thermocouple trees)	Each set 0.10, 0.20, 0.51, 0.81, 1.12, and 1.73 m
	Differential pressure array (pressure probes)	0.20, 0.41, 0.61, 0.81, 1.02, 1.22, 1.42, 1.63, 1.83, 2.03, and 2.24 m
	Gas velocity array (bi-directional velocity probes)	0.30, 0.91, 1.22, 1.52, and 1.83 m
Exhaust hood	Gas temperature array	Nine positions evenly spaced
	Gas velocity array	Nine position evenly spaced
	Gas concentration, CO, CO_2_, and O_2_	At centerline of hood
	Smoke obscuration	At centerline of hood

a Notation used for rooms and vents were changed from the original report to be consistent throughout this report. For reference, names used in the original report are shown in parentheses.

b Notation used for instrumentation was changed from the original report to be consistent throughout this report. For reference, names used in the original report are shown in parentheses.

c Distances are measured from floor.

**Table 6:** Room fire tests

Test	Furnishings	Wall material^a^	Sprinkler	Test durations	Ambient room conditions	
					Temperature °C	Relative humidity %
R1	Std. set	12.7 mm gypsum board	None	1800	23	50
R4			Dry	1800	23	56
R6			Dry	1800	23	45
R2		6.4 mm A/D plywood over 12.7 mm gypsum board	Dry	525	23	50
R3			Wet	470	22	52
R5			Dry	1800	24	48

a Ceiling material was 15.9 mm fire-resistant gypsum board.

**Table 7:** Open burn tests

Test	Furnishings	Wall behind headboard	Test durations	Ambient room conditions	
				Temperature °C	Relative humidity %
O4	Std. set	No wall	1800	22	32
O6			1800	21	38
O1		12.7 mm gypsum board	1800	22	50
O3			1800	21	40
O2		6.4 mm A/D plywood over gypsum board	1800	22	50
O5			1800	21	38

**Table 8:** Fuel loading in fire tests

Fuel item	Combustible weight, kg	
	Open burns	Room burns
Mattress and box spring^a^	24.7	24.7
Headboard	14.4	14.4
Night table	10.6	10.6
Bedding	3.2	3.2
Filled wastebasket	0.75	0.75
Total combustible furnishings	53.7	53.7
Plywood^b^	19.5	77.9

a Mattress and box spring weight excluding that of the inner springs.

b Only used for open burn tests 2 and *5* and for room tests 2, 3, and 5.

**Table 9:** Wastebasket ignition source

Wastebasket—polyethylene wastebasket weight: 0.34 kg
Trash contents, in order of stacking
1 polyethylene liner
16 sheets of newspaper
1 paper cup, 3 oz, crumpled
2 sheets of writing paper
3 paper tissues, crumpled
1 cigarette pack, crumpled
1 milk carton, 8 oz
2 paper cups, crumpled
1 cigarette pack, crumpled
1 sheet of writing paper, crumpled
2 paper tissues, crumpled
Total weight of contents: 0.41 kg

**Table 10:** Room and vent sizes for three room tests with a corridor

Location^a^	Room	Vent	Dimensions (m)^b^
Room 1 (first room)	✓		2.34 × 2.34 × 2.16
Doorway, room 1 to room 2^c^ (first room doorway)		✓	0.81 × 1.60 × 0.53
Room 2 (second room)	✓		2.44 × 12.19 × 2.44
Doorway, room 2 to room 3^d^ (third room doorway)		✓	0.79 × 2.04 × 0.40
Doorway, room 2 to ambient (second room exit doorway)		✓	0.76 × 2.03 × 0.41
Room 3 (third room)	✓		2.24 × 0.94 × 2.43

a Notation used for rooms and vents were changed from the original report to be consistent throughout this report. For reference, names used in the original report are shown in parentheses.

b For rooms, dimensions are width × depth × height. For vents, dimensions are width × height × soffit depth.

c Doorway from room 1 to room 2 was actually 1.02 × 1.03 × 2.00 m passageway.

d Doorway from room 2 to room 3 was actually 0.79 × 0.94 × 2.04 m passageway.

**Table 11:** Construction materials

	Location	Material	Thickness mm	Densitykg/m^3^	Heat capacity KJ/kg·K	Thermal conductivity W/m·K	Emissivity
Room 1	Wall substrate	Fire brick	113	750	1.04	0.36/200 °C	0.80
						0.38/300 °C	
						0.45/600 °C	
	Ceiling substrate	Calcium silicate			Same as room 2 walls		
	Walls and ceiling^a^	Ceramic fiber	50	128	1.04	0.09/300 °C	0.97
						0.17/600 °C	
						0.25/900 °C	
	Floor^a^	Fire brick			Same as wall substrate		
Room 2	Ceiling and wall substrate	Gypsum board	12.7	930	1.09	0.17	
	Ceiling and walls^a^	Calcium silicate	12.7	720	1.29/200 °C	0.12/200 °C	0.83
					1.33/300 °C	0.11/300 °C	
					1.55/600 °C	0.12/600 °C	
	Floor substrate	Concrete	102	2280	1.04	1.8	
	Floor^a^	Gypsum board	12.7	930	1.09	0.17	
Room 3	Walls and ceiling^a^	Gypsum board^b^	12.7	930	1.09	0.17	
	Floor^a^	Concrete	102	2280	1.04	1.8	

a Interior finish.

b Gypsum board over studs.

**Table 12:** Location of instrumentation for three room tests with a corridor

Room location^a^	Measurement type^b^	Position^c^
Room 1 (first room)	Gas temperature array (tree 1)	0.15, 0.36, 0.66, 0.97, 1.27, 1.88, 2.03, and 2.15 m
	Surface temperature	Four walls (0.55 and 1.64 m), ceiling, and floor
Doorway, room 1 to room 2 (first room doorway)	Gas temperature array (tree 2)	0.15, 0.30, 0.61, 0.91, 1.22, and 1.52 m
	Differential pressure array (static pressure probes)	25 mm, 0.30, 0.61, 1.22, and 1.52 m
Room 2 (second room)	Three gas temperature arrays (trees 3, 4, and 5)	0.15, 0.30, 0.61, 0.91, 1.22, 1.52, 1.83, 2.13, 2.29, 2.44 m
	Surface temperature	0.61 and 1.83 m
	Smoke obscuration	Horizontal array at 0.61, 0.91, 1.22, 1.52, 1.83 and 2.29 m
		Vertical measurement
Doorway, room 2 to room 3 (third room doorway)	Gas temperature array (tree 7)	0.15, 0.61, 0.91,1.07,1.22, 1.52, 1.83, and 1.93 m
	Differential pressure (static pressure probes)	80 mm
Doorway, room 2 to ambient (second room exit doorway)	Gas temperature array (tree 6)	0.15, 0.30, 0.61, 1.22, 1.52, 1.83, and 2.13 m
	Differential pressure array (static pressure probes)	76 mm, 0.61, 1.22, 1.52, and 1.83 m
Room 3 (third room)	Gas temperature array (tree 8 in third room)	0.15, 0.61, 0.91, 1.07, 1.22, 1.52, 1.83, 2.13, 2.29, and 2.44 m
Exhaust hood	Gas temperature array	Nine positions evenly spaced
	Gas velocity array	Nine position evenly spaced
	Gas concentration, CO, CO_2_, and O_2_	At centerline of hood
	Smoke obscuration	At centerline of hood

a Notation used for rooms and vents were changed from the original report to be consistent throughout this report. For reference, names used in the original report are shown in parentheses.

b Notation used for instrumentation was changed from the original report to be consistent throughout this report. For reference, names used in the original report are shown in parentheses.

c Distances are measured from floor.

**Table 13:** Initial conditions for tests with gas burner (third room closed)

Test	Room 2 doorway to ambient	${\dot{Q}}_{T}$^a^ kW	Smoke^b^ source	*P*_a_^c^ mm Hg	*T*_a_^d^ °C	*T*_out_^e^ *°*C	RH^f^ %	Room 2 lights
100 A	open	100	C					on
100 B				756	23		68	
100 C				751	23		72	
100 D			none	749	23		74	
100 E			C	760	21		58	
100 G			A	764	22		54	
100 H								off
100 I				760	20	−7	46	
100 J				755	21	2	51	
100 L	closed			751	21	2		g
100 M				749	21	4	41	off
100 N				745	21	6	45	on
100 O				740	21	11	45	g
300 A	open	300		744	21	12	63	on
300 B				754	21	8	54	off
300 C								
500 A		500	none	756	22	13	57	
500 B			A	748	22	10	50	
500 C								g
500 D				768	21	−6	31	on
500 E				748	21	4		off
500 F				755	21	6	46	

a Calculated gas burner heat release rate (includes acetylene).

b C is for candle; A is for acetylene.

c Ambient barometric pressure in test facility.

d Ambient temperature in test facility at the start of the test.

e Ambient outside temperature at the start of the test.

f Relative humidity in test facility at the start of the test.

g Uncertain.

**Table 14:** Initial conditions for tests with gas burner (third room open)

Test	Room 2 doorway to ambient	${\dot{Q}}_{T}$^a^ kW	Smoke^b^ source	*^P^*_a_^c^ mm Hg	*T*_a_^d^ °C	*T*_out_^e^ °C	RH^f^ %	Room 2 lights	Footnotes
100 U	open	100	A	761	22	16	66	off	
100 V				747	20	2	43		
100 W				744	21	1	48		
100 X				752	23	20	61		
100 Y				751	23	26	58		
100 Z				754	22	18	61		
100 AA				752	22	30	62		
100 AB				754	22	26	55	on	
100 P	closed			749	22	24	70	off	
100 Q				749	21	21	63		g
100 R				748	22	26	67		g
100 S				749	22	30	65		g
300 F	open	300		748	22	24	70		g
300 G				754	23	19	65		
300 H				741	23	26	66		
300 D	closed			748	22		66		g,h
300 E				748	22	26	70		g,h
500 G	open	500		750	22	24	68		g

a Calculated gas burner heat release rate (including acetylene) —accuracy within 2%.

b A is for acetylene.

c Ambient barometric pressure in test facility at the start of the test.

d Ambient temperature in test facility at the start of the test.

c Ambient outside temperature.

f Relative humidity in test facility.

g Anemometer used to measure flow in third room doorway.

h Burner flames prematurely extinguished by ultraviolet sensor.

**Table 15:** Room and vent sizes for four room tests with a corridor

Location^a^	Room	Vent	Dimensions (m)^b^
Room 1 (burn room)	**✓**		3.64 × 3.63 × 2.45
Doorway, room 1 to room 2 (burn room doorway)		**✓**	0.92 × 2.05 × 0.40
Window, room 1 to ambient (burn room window)^c^		**✓**	0.85 × 0.85 × 0.34
Room 2 (corridor)	**✓**		2.43 × 18.89 × 2.43
Doorway, room 2 to room 3 (target room 1 doorway)		**✓**	0.88 × 202 × 0.43
Doorway, room 2 to room 4 (target room 2 doorway)		**✓**	0.88 × 2.02 × 0.43
Room 3 (target room 1)	**✓**		3.64 × 3.65 × 2.45
Room 4 (target room 2)	**✓**		3.65 × 3.65 × 2.43

a Notation used for rooms and vents were changed from the original report to be consistent throughout this report. For reference, names used in the original report are shown in parentheses.

b For rooms, dimensions are width × depth × height. For vents, dimensions are width × height × soffit depth.

c Closed during some tests.

d An observation alcove measuring 1.28 × 0.86 × 1.99 m was located in the corner of room 4 reducing the total volume of the room.

**Table 16:** Location of instrumentation for four room tests with a corridor

Room location^a^	Measurement type^b^	Position^c^
Room 1 (burn room)	Gas temperature array (station 9)	0.26, 0.66, 1.07, 1.48, 1.88, 2.19, 2.34, and 2.39 m
	Surface temperature	Ceiling
	Gas concentration, CO, CO_2_, and O_2_	1.48 m
	Smoke obscuration array	0.26, 1.07, 1.88, and 2.39 m
	Differential pressure (wall pressure taps)	Two locations at 2.05 m
Doorway, room 1 to room 2	Differential pressure (wall pressure taps)	2.05 m
Room 2 (corridor)	Gas temperature arrays (stations 2–7) 3, 4, and 5)	0.26, 0.66, 1.07, 1.48, 1.88, 2.19, 2.34, and 2.39 m
	Surface temperatures (stations 3, 5, and 7)	Ceiling at three locations
	Gas concentration, CO, CO_2_, and O_2_ (station 4)	Center of corridor, CO, CO_2_, and O_2_ at 1.48 m, CO_2_ at 0.26 and 2.39 m
	Smoke obscuration arrays (stations 2, 4, and 6)	Three locations at 0.26, 1.07, 1.88, and 2.39 m
	Differential pressure (wall pressure taps)	Corridor end near room 4 at 2.05 m
Doorway, room 2 to room 3	Differential pressure (wall pressure taps)	2.05 m
Room 3 (target room 1)	Gas temperature array (station 8)	0.26, 0.66, 1.07, 1.48, 1.88, 2.19, 2.34, and 2.39 m
	Gas concentration, CO, CO_2_	1.48 m
	Smoke obscuration array	0.26, 1.07, 1.88, and 2.39 m
Room 4 (target room 2)	Gas temperature array (station 1)	0.26, 0.66, 1.07, 1.48, 1.88, 2.19, 2.34, and 2.39 m
	Gas concentration, CO, CO_2_	1.48 m
	Smoke obscuration array	0.26, 1.07, 1.88, and 2.39 m
	Differential pressure (wall pressure taps)	2.05 m

a Notation used for rooms and vents were changed from the original report to be consistent throughout this report. For reference, names used in the original report are shown in parentheses.

b Notation used for instrumentation was changed from the original report to be consistent throughout this report. For reference, names used in the original report are shown in parentheses.

c Distances are measured from floor.

**Table 17:** Calibration of 031 m diameter propylene burner

Orifice normal no. of flow units	Orifice actual no. of flow units	${\dot{Q}}_{t}$(kW)	$\frac{{\dot{Q}}_{c}}{{\dot{Q}}_{t}}$	$\frac{{\dot{m}}_{\mathrm{CO}}}{{\dot{m}}_{{\mathrm{CO}}_{2}}}$	$\frac{{\dot{m}}_{p}}{{\dot{m}}_{f}}$
1	0.97	25.6			
2	2.10	56.5	0.60	0.0105	0.083
3	3.07	86.3	0.60	0.0095	0.076
4	3.91	105.2	0.57	0.0094	0.071

**Table 18:** Experimental conditions for steady state fires

Test	Burner diam.	HRR	Doors (rooms 3&4)	Forced vent	Room 1 door	Room 1 window	Room 2	
							Partition	Ceiling vent
1	0.30 m	56 kW	closed	none	open	closed	no	none
2								
3						open		
4					closed	closed		
5						open		
6					open	closed		
7			open					
8								
9					closed			
10						open		
11					open			
12			closed	18 g/s		closed		
13				9 g/s				
14					closed			
15				18 g/s				
16	0.91 m	522 kW		none	open			
17								
18						open		
19			open					
20			closed		closed			
21			open		open	closed		
22					closed			
23			closed	36 g/s	open			
24				72 g/s				
25				144 g/s				

**Table 19:** Experimental conditions for growing fires

Test	Burner diam.	HRR	Doors (rooms 3&4)	Forced vent	Room 1 door	Room 1 window	Room 2	
							Partition	Ceiling vent
26	0.91 mm	240 s	closed	none	closed	open	no	none
27					open	closed		
28		120 s						
29					closed	open		
30			open		open	closed		
31		240 s						
32			closed			open		
33			open					
34		120 s						
35		240 s			closed	closed		
36		120 s	closed		open	open		
37			open		closed	closed		
48		240 s			open	closed	no	
49								
50			closed		closed	open		
51			open		open	closed		
52			closed		closed	open		
53								
54								
55			open		open	closed		
56								
57		120 s						
58		60 s						
59		480 s						

**Table 20:** Experimental conditions for tests with corridor partition

Test	Burner diam.	HRR	Doors (rooms 3 & 4)	Forced vent	Room 1 door	Room 1 window	Room 2	
							Partition	Ceiling vent
38	0.91 m	240 s	open	none	open	closed	yes	none
39								natural
40		120 s						
41		240 s						170 g/s return
42						open		natural
43								170 g/s return
44		120 s						natural
45		240 s				closed		170 g/s supply
46						open		
47								none

**Table 21:** Room and vent sizes for multiple story building tests

Location^a^	Room	Vent	Dimensions (m)^b^
Room 1 (2nd floor burn room)	**✓**		6.2 × 6.2 × 2.3
Doorway, room 1 to room 2 (2nd floor burn room doorway)		✓	0.78 × 2.1 × 0.22
Window, room 1 to ambient^c^ (burn room window)		**✓**	1.2 × 1.2 × 0.37
Room 2 (corridor)	**✓**		1.9 × 14.6 × 2.6
Doorway, room 2 to room 3		**✓**	0.78 × 2.1 × 0.5
Doorway^d^, room 2 to room 4 (2nd floor corridor to stairwell)		**✓**	0.016 × 2.1 × 0.5, tests 1−3 0.03 × 2.1 × 0.5, tests 4−12
Room 3 (remainder of 2nd floor)	**✓**		10.3 × 10.3 × 2.7
Rooms 4, 5, 6, 7, 8, 9, and 10 (basement, 1st, 3rd, 4th, 5th, 6th, and 7th floors)	**✓**		13.3 × 13.3 × 2.6
Doorways^d^, rooms 4, 5, 6, 7, 8, and 9 to room 11 (basement, 1st, 3rd, 4th, 5th, and 6th floors to stairwell)		**✓**	0.03 × 2.1 × 0.5
Doorway^d^, room 10 to room 11 (7th floor to stairwell)		**✓**	0.016 × 2.1 × 0.5, tests 1−3, 12 0.91 × 2.1 × 0.5, tests 4−11
Room 11 (stairwell)	**✓**		3.0 × 4.1 × 21.5
Doorway^d^, room 11 to ambient (stairwell to outside at basement)	**✓**		0.016 × 2.1 × 0.5, tests 1−5, 10−11 0.91 × 2.1 × 0.5, tests 6−9, 12

a Notation used for rooms and vents were changed from the original report to be consistent throughout this report. For reference, names used in the original report are shown in parentheses.

b For rooms, dimensions are width × depth × height. Room dimensions are approximate. Only total volume and ceiling heights were noted in the original report. For vents, dimensions are width × height × soffit depth.

c Only for test 12.

d An average value is used for leakage areas around doors to stairwell. Other leakage paths from floors were noted in the report but not quantified (elevator shaft, building wiring, spiral stairs). These may need to be estimated to obtain accurate model predictions.

**Table 22:** Location of instrumentation for multiple story building tests

Room location^a^	Measurement type^b^	Position^c^
Room 1 (2nd floor burn room)	Gas temperature array (thermocouple tree) Specimen mass loss	61 mm, 0.46, 0.91, 1.37, 1.83, and 2.28 m
Room 2 (corridor)	Gas temperature arrays (thermocouple trees)	Two near burn room at 61 mm, 0.48, 0.96, 1.44, 1.92, and 2.39 m near elevator at 61 mm, 0.52, 1.05, 1.57, 2.09, and 2.61 m
	Gas concentration, CO, CO_2_, and O_2_ (gas analysis)	Center of corridor, at 1.52 m
	Smoke obscuration (smoke meter)	Center of corridor, at 1.52 m
	Differential pressures (pressure differences)	Through floor to room 6 and through ceiling to room 7
Doorway, room 2 to room 11 (2nd floor corridor to stairwell)	Differential pressures (pressure differences)	0.15 and 2.2.3 m
Rooms 4, 5, 6, 7, 8, 9, and 10 (basement, 1st, 3rd, 4th, 5th, 6th, and 7th floors)	Gas temperature array (thermocouple tree)	61 mm, 0.65, 1.31, 1.96, and 2.61 m
	Gas concentration, CO, CO_2_, and O_2_ (gas analysis)	Test 3, in room 6, 1.52 m test 5, 9, 10, and 11, in room 10, 1.52 m
	Smoke obscuration (smoke meter)	In rooms 6 and 10, 1.52 m
Doorways, rooms 4, 7, and 10 to room 11, (basement, 4th, and 7th floor to stairwell)	Differential pressure (pressure difference)	1.52 m
Room 11 (stairwell)	Gas temperature array (thermocouple trees on each floor)	61 mm, 0.7, 1.4, 2.0, 2.7, 2.8, 3.4, 4.0, 4.7, 5.4, 5.5, 6.1, 6.7, 6.8, 7.4, 8.1, 8.2, 8.7, 9.4, 10.1, 10.8, 10.9, 11.4, 12.1, 12.8, 13.5, 13.6, 14.1, 14.8, 15.5, 16.1, 16.2, 16.8, 17.5, 18.2, 18.8, 18.9, 19.5, 20.2, 20.9, and 21.5 m
	Differential pressures to ambient (pressure differences)	1.5, 12.3, and 20.4 m
	Smoke obscuration array	0.26, 1.07, 1.88, and 2.39 m
Ambient	Gas temperature, wind velocity and wind direction	

a Notation used for rooms and vents were changed from the original report to be consistent throughout this report. For reference, names used in the original report are shown in parentheses.

b Notation used for instrumentation was changed from the original report to be consistent throughout this report. For reference, names used in the original report are shown in parentheses.

c Distances are measured from floor.

**Table 23:** Experimental conditions

Test	Test type	Fire load^b^ kg	Zoned smoke control^c^	Stairwell pressurization^d^	Activation time^e^ min	Condition of doors in Room 11 to		
						Ambient	Room 2	Room 10
1	wood fire	136	off	off		closed	closed	closed
2	smoke bomb		on	″	0	″	″	″
3	wood fire	91	″	″	0	″	″	″
4	smoke bomb		off	″		″	$\frac{1}{2}$ inch	open
5	wood fire	136	″	″		″	″	″
6	smoke bomb		on	on	0	open	″	″
7	wood fire	136	″	″	0	″	″	″
8	smoke bomb		″	″	4	″	″	″
9	wood fire	136	″	″	4	″	″	″
10	sprinklered	136	off	off		closed	″	″
11	sprinklered	136	″	″		″	″	″
12	wood fire	272	on	on	0	open	″	closed

a All fires in the second floor corridor and all windows closed, except for test 12, where the fire was in the fire-hardened room on the second floor, and the window in that room was open.

b Fire load is approximate.

c Zoned smoke control consisted of pressurization of first and third floors at 0.94 m^3^/s each, and the exhaust of the second floor at the same rate.

d Stairwell pressurization consisted of supplying 3.3 m^3^/s into the stairwell at the first floor with the exterior basement door open.

e Activation time is the time after ignition that the smoke control system and stairwell pressurization system are turned on.

f Second floor door designation $\frac{1}{2}$ in indicates that the door was cracked open $\frac{1}{2}$ in.

## Footnotes

1 Certain commercial equipment, instruments, or materials are identified in this paper to specify adequately the experimental procedure. Such identification does not imply recommendation or endorsement by the National Institute of Standards and Technology, nor does it imply that the materials or equipment identified are necessarily the best available for the purpose.

## Glossary

Δ*p*: pressure difference (Pa)
*σ*: average specific extinction area (m^2^/kg)
*C*: opening flow coefficient (kg^1/2^m^1/2^K^1/2^. Typical values are 0.73 for outflow and 0.68 for inflow.
*C_N_*: empirically determined value (typical value is 0.2)
*E*: net heat released by complete combustion per unit of oxygen consumed (kJ/kg of O_2_). Typical values are 13100 KJ/kg for organics, 17600 kJ/kg for combustion of CO to CO_2_
*g*: gravitational constant (9.81 m/s^2^)
*h*: height (m)
*I*: beam intensity
*k*: smoke extinction coefficient (m^−1^)
*L*: measurement path length for the smoke (m)
*m*: mass (kg)
$\dot{m}$: mass flow rate (kg/s)
*M*: molecular weight (kg/kmol). Typical value for air is 28.95 kg/kmol
$\dot{q}$: rate of heat released from the fire room (kW)
*p*: pressure (Pa). Ambient value is 101325 Pa
*P*: total smoke production (m^2^)
*R*: universal gas constant (8314 J/kg mol K)
*t*: time (s)
*T*: gas temperature (K)
*υ*: gas velocity (m/s)
$\dot{V}$: volume flow rate (m^3^/s)
*W*: opening width (m)
*X*: measured concentration (mole fraction)

**Subscripts used in nomenclature**
∞: final value
a: air
b: bottom
CO: carbon monoxide
CO_2_: carbon dioxide
d: doorway
dry: dry air
e: exhaust duct
f: fuel
h: horizontal
H_2_O: water
i: inside room
l: lower
max: maximum
N: at neutral plane or layer interface height
o: outside room or initial value
O_2_: oxygen
t: top
u: upper
v: vertical

**Subscripts used in nomenclature**
o: ambient
